# Quantum thermodynamics with a single superconducting vortex

**DOI:** 10.1126/sciadv.ado4032

**Published:** 2024-07-31

**Authors:** Marek Foltyn, Konrad Norowski, Alexander Savin, Maciej Zgirski

**Affiliations:** ^1^ Institute of Physics, Polish Academy of Sciences, Aleja Lotnikow 32/46, Warsaw PL 02668, Poland.; ^2^ QTF Centre of Excellence, Department of Applied Physics, Aalto University, Aalto FI-00076, Finland.

## Abstract

We demonstrate complete control over dynamics of a single superconducting vortex in a nanostructure, which we coin the Single Vortex Box. Our device allows us to trap the vortex in a field-cooled aluminum nanosquare and expel it on demand with a nanosecond pulse of electrical current. Using the time-resolving nanothermometry we measure $4\cdot 10^{-19}$ joules as the amount of the dissipated heat in the elementary process of the single-vortex expulsion. Our experiment enlightens the thermodynamics of the absorption process in the superconducting nanowire single-photon detectors, in which vortices are perceived to be essential for a formation of a detectable hotspot. The demonstrated opportunity to manipulate a single superconducting vortex reliably in a confined geometry comprises a proof of concept of a nanoscale nonvolatile memory cell with subnanosecond write and read operations, which offers compatibility with quantum processors based either on superconducting qubits or on rapid single-flux quantum circuits.

## Teaser

Electric current pulses enable fully controllable manipulation of a single superconducting vortex in a nanostructure.

## INTRODUCTION

Thermodynamics involves studies of the heat flow arising from the difference in temperature between two bodies, as stated in the second law. When this flow is considered at a single undividable particle level, we investigate the thermodynamics at its natural microscopic limit governed by quantum physics (*1*). These studies are preferably performed in nanoscale devices cooled down to the lowest temperature where the quantum effects can flourish and temperature gradients can be set on demand. The seminal experiments performed in the field involve demonstrations of quantized thermal conductance of heat not only by single modes of phonons (*2*) and photons (*3*, *4*) but also by single-electron channels (*5*) and anyons (*6*). Besides the steady-state investigations, researchers were able to demonstrate the control of the heat transport at a single-particle level as exemplified in the experiment with an electron turnstile (*7*). The laws of thermodynamics first written down in 19th century, owing to their only statistical validity, do not need to hold for microscopic systems that exchange quantized amount of energy, e.g., it is possible to observe heat flow from colder to hotter object albeit with lower probability than in the opposite direction favored by the second law of thermodynamics (*1*, *8*). The monitoring and control of the heat transport at a single-particle level allowed researchers recently to revive the Maxwell demon, who, for a long time, seemed to be only an intellectual curiosity (*9*, *10*). The experimental verification of the Landauer’s principle linking the erasure of a single bit of information with the minimum amount of dissipated heat of *k*_B_*T*ln (*2*) (i.e., the Landauer bound) exorcised the demon and connected two worlds: the information theory and thermodynamics (*11*).

The recent advancements in experimental techniques, particularly in nanothermometry, have allowed to shine a new light on various frequently studied quantum phenomena, in which the role of dissipation had been only postulated, sometimes a priori neglected, but never verified experimentally. Researchers were able to perform thermal imaging of a graphene with SQUID-on-tip and found the dissipation in resonant states along the edges of the sample (*12*). The other team measured a pronounced temperature rise in a nanoscopic metallic island serving as a junction in an RF-SQUIPT due to a single-phase slip event (*13*). Similar experiments are expected to deeply affect our understanding of the dynamics of the quantum systems, in which dissipation is responsible for the loss of the quantum coherence or suppression of topological protection.

It is the aim of our presentation to appoint to the field of quantum thermodynamics a new actor—the superconducting vortex. It appears naturally in type II superconductors upon exceeding a certain magnetic field as an energetic compromise between Meissner state (when magnetic field is expelled from the sample) and normal state (when magnetic field can entirely pass through the sample). Superconducting vortex is a pure quantum object: It is microscopic ring of supercurrent, which collects 2π of superconducting phase on one round trip and encircles quantized filament of magnetic flux, known as flux quantum Φ_0_ = *h*/2*e*. The vortex is thus the simplest topologically protected pattern of the quantized circulation to provide partial screening of the external magnetic field while avoiding the entry or exit of the current lines into or from the superconductor. As long as superconducting vortices do not move, the externally applied current *I*_A_ is dissipationless, for it finds its way between vortices and preserves perfect conductivity. However, as *I*_A_ is increased, the Lorentz force acting on vortices may put them into motion. Mobile vortices become source of dissipation: Each moving vortex excites quasiparticles (QPs) along its trajectory (*14*). For fast electron-electron interaction, the presence of excess QPs is thermodynamically equivalent to the elevation of electron temperature (*15*, *16*).

In our work, we trap and expel a single vortex on demand with pulses of electrical current. The supreme control over single-vortex dynamics combined with the time-resolved nanothermometry (*17*) allows us to measure the electron temperature jump after vortex has been expelled from the aluminum nanoscale sample and the subsequent thermal relaxation. We get the experimental access to the energetic cost of a single-vortex expulsion. Apart from a deep insight into thermodynamics of a moving vortex, we present an experimental platform for emerging field of vortex electronics (*18*–*21*). Our device not only is a simple memory cell but also shows features of a superconducting diode (fig. S1).

Our study may improve understanding of the detection mechanism of superconducting nanowire single-photon detectors (*22*–*24*) and transition-edge sensors (*25*). It suggests that moving vortex in these devices could not only amplify signal after the initial photon absorption by producing additional dissipation (fig. S2B) but also be a source of dark counts (*14*).

**Fig. 1.**
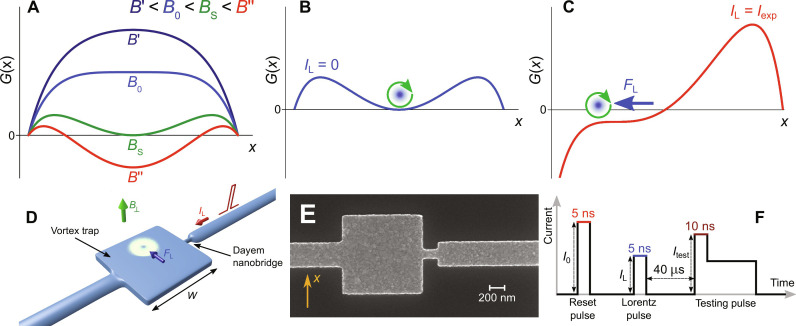
Single Vortex Box (SVB). (**A**) Landscape of the Gibbs free energy of a single-vortex state across the width of the box at various magnetic fields *B*_⊥_ and with no applied current *I*_L_. For *B*_⊥_ > *B*_0_, the state with vortex becomes energetically favorable when sample is cooled across *T*_c_. (**B** and **C**) The effect of the applied current on the tilt of the potential energy. For *I*_L_ = *I*_exp_, the dependence shows no minimum that would stabilize the vortex, and it leaves the sample pushed out by the Lorentz force *F*_L_. *I*_exp_ grows with the field because vortex is stronger bound in local energy minimum further away from the transition field *B*_0_. (**D**) Layout of the studied nanostructure consisting of an SVB, a Dayem nanobridge, and connecting leads. The Lorentz force exerted on the vortex by the applied current *I*_L_ in the presence of perpendicular magnetic field *B*_⊥_ is depicted schematically. (**E**) Scanning electron microscopy image of the working aluminum device. (**F**) The pulse protocol used in the experiment.

Superconductor expels the externally applied magnetic field from its interior, owing to existence of Meissner screening currents. If the kinetic energy of these currents becomes too large, then it is energetically favorable for the sample to let some magnetic field lines in. Magnetic flux that enters into sample involves formation of quantized loops of supercurrent. If the sample is small enough and cooled across critical temperature *T*_c_ in applied magnetic field, it is possible to trap just a single vortex, provided that Gibbs free energy develops a metastable minimum (Fig. 1A and text S1) (*26*, *27*). For superconducting strips of width *W*, this minimum is separated with the Bean-Livingston barriers from the edges of the strip and is first established when magnetic field exceeds the threshold value, i.e., $B_{0}∼\frac{\pi \Phi_{0}}{4W^{2}}$ (*28*–*30*). This prediction holds for superconducting strips (*31*) but qualitatively (up to a numerical factor of the order of unity) is also correct for the squared zero-dimensional (0D) confinements that are studied below (*32*). The presented model also predicts that the trapped vortex can be expelled from the nanostructure by application of the pulse of electrical current, which tilts the potential energy and removes the local energy minimum: Because of the Lorentz force, the vortex is pushed to the side of the square, and, eventually, it escapes out of the sample (Fig. 1, C and D). Once the current pulse is over, the potential regains its original shape with minimum in the middle, but the vortex is not present in the nanostructure. Because of the increased depth of the potential well for larger fields, they implicate higher currents necessary to expel the vortex.

The effects related to dissipation due to moving vortices were widely studied in current-driven thin superconducting films (*33*–*35*). The investigations were performed for samples containing huge number of vortices moving in steady states, and the effect of dissipation was deduced from voltage appearing on the sample once the current bias threshold for the vortex movement was exceeded. Researchers identified flux flow regime (*36*) and avalanche regime (*37*–*39*), but the carried out experiments did not give direct access to the elementary dissipative process, which is the expulsion of a single vortex from a superconductor. The theoretical prediction assumes that vortex moving in the strip excites QPs along its path (*14*, *40*). It is this hypothesis that we verify in the demonstrated experiment. We provide calorimetric measurement of the thermal footprint left behind the expelled vortex.

## RESULTS

We fabricate Single Vortex Box (SVB) with standard e-beam lithography by evaporating 30 nm of aluminum (Fig. 1E), which guarantees type II superconducting behavior of our device (text S2). The SVB is attached to a short Dayem nanobridge, whose critical current is sensitive to the vortex state of the box (*32*). The structure (box + nanobridge) is connected to the contact pads through 15-μm-long and 300-nm-wide leads. This geometry, although very simple, allows not only to monitor but also to manipulate the vortices in the box with pulses of electrical current. We can initialize the box in a single-vortex state (with the reset pulse), expel the vortex (with the Lorentz pulse), and detect the presence or the absence of the vortex by probing the switching current of the nanobridge with the testing pulse (see pulse protocol in Fig. 1F and Materials and Methods for details). Our approach is also compatible with time-resolved switching thermometry developed in recent years (*15*); therefore, we can measure the thermal response of the trap after applying a current pulse, which changes the vortex state of the box (figs. S3 to S5).

### Detection of a single vortex

**Fig. 2.**
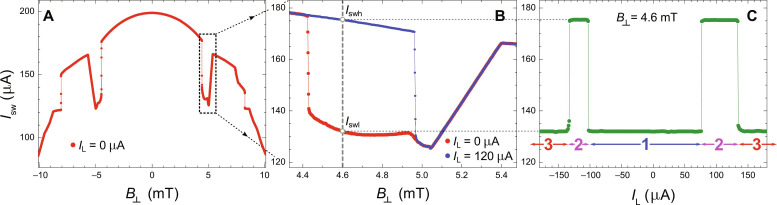
Electrical probing and manipulation of the vortex state. (**A**) Switching current of the nanobridge versus perpendicular magnetic field *I*_sw_(*B*_⊥_) reveals a pronounced dip in the characteristics for the field values where the entry of a single vortex is expected. Here, only the reset and testing pulses are used (cf. Fig. 1F). (**B**) *I*_sw_(*B*_⊥_) dependence in the region of the dip. The red curve is a detailed measurement of the region of the suppressed switching current *I*_swl_ visible in the curve of (A) inside the dashed frame, and the blue curve presents the effect of the application of the additional pulse, called the Lorentz pulse (cf. Fig. 1F). It is high enough to expel the vortex but too low to switch the bridge. The following testing pulse probes the box in the Meissner state: This time, there is no vortex to be expelled. Consequently, there are no QPs excited in the box, and the switching current remains at its high value *I*_swh_. (**C**) Switching current of the nanobridge as a function of the Lorentz pulse amplitude recorded at the fixed magnetic field in the dip region [dashed vertical line in (B)]. For low values of the Lorentz pulse (region 1), it can neither expel vortex nor switch the bridge. In region 2, Lorentz pulse can expel the vortex but not switch the bridge. Last, for the highest values of the Lorentz pulse (region 3), it first expels the vortex and then switches the bridge. The bath temperature is *T*_0_ = 400 mK.

We measure switching current of the nanobridge as a function of the perpendicular magnetic field *I*_sw_(*B*_⊥_) (Fig. 2A) using the pulse sequence presented in Fig. 1F with *I*_L_ = 0. At low values of the applied field, we see continuous suppression of the critical current due to enhancement of the Meissner screening currents expelling away magnetic field lines. This regime is followed by narrow range of fields where we observe the pronounced dip in the *I*_sw_(*B*_⊥_) characteristics, being a sign of strong reduction of the superconducting order parameter due to dissipation. The abrupt transition corresponds to the first vortex penetration field $B_{0}∼\frac{\pi \Phi_{0}}{4W^{2}}$ . The trace is thus consistent with the interpretation in which we expel the vortex from nanostructure in the narrow field window. In the discussed case, the testing pulse of the bridge also provides the Lorentz force necessary to get rid of the vortex. The leaving vortex produces the excess population of QPs, which suppresses the switching current of the bridge. We see that at higher fields, *I*_sw_ recovers to a big extent, signaling absence of the dissipative process due to the moving vortex. It happens because the current needed to expel the vortex *I*_exp_ grows with field and becomes larger than *I*_sw_. The *I*_sw_(*B*_⊥_) characteristics for other samples are provided in fig. S6.

### Manipulation of a single vortex

It is possible to partially “heal” the dip, i.e., extend the Meissner state of the box into the higher magnetic fields by the application of the Lorentz current pulse, which expels the vortex but does not switch the junction (Fig. 2, A and B; see the difference for *I*_L_ = 0 and *I*_L_ = 120 μA). The testing pulse that probes the bridge is then applied a long time after expulsion, when the box comes back to thermal equilibrium and finds the bridge in the Meissner state corresponding to a high value of the switching current *I*_swh_. This scenario is presented for *I*_sw_(*B*_⊥_) dependence in Fig. 2B. By scanning the amplitude of the Lorentz pulse at a fixed magnetic field inside the cusp, one can find three regions (Fig. 2C). In the first one, for the lowest values of the Lorentz pulse, the switching current is also low (*I*_swl_). Here, the Lorentz current cannot remove the vortex, but the testing pulse provides enough amplitude to do it. It first expels the vortex at its rising slope [the rising time of the pulse is equal to 2 to 3 ns, i.e., its dynamics is much slower than that of the vortex (*14*)]. Following the expulsion, the box warms up, and as a consequence, the same testing pulse probes the thermally excited state of the bridge. It results in a low value of the switching current *I*_swl_ = *I* (no vortex, *T*_0_ + Δ*T*). In the second region, the Lorentz pulse expels the vortex, but it does not switch the bridge. The box is now in the Meissner state. The testing pulse arriving 40 μs later finds the SVB well equilibrated at the bath temperature *T*_0_ = 400 mK, and we find a high value of the switching current *I*_swh_ = *I* (no vortex, *T*_0_). Last, in the third region, the Lorentz pulse expels the vortex, but because of the too high amplitude, it also switches the bridge. The bridge and box go to the normal state, and in the following cooldown, another vortex is trapped in the box. The Lorentz pulse works here as the second reset pulse and overall does not change the state of the box. When testing pulse arrives, it finds the vortex in the box, and the expulsion-switching scenario described for the region 1 follows: Switching current is low again (*I*_swl_).

**Fig. 3.**
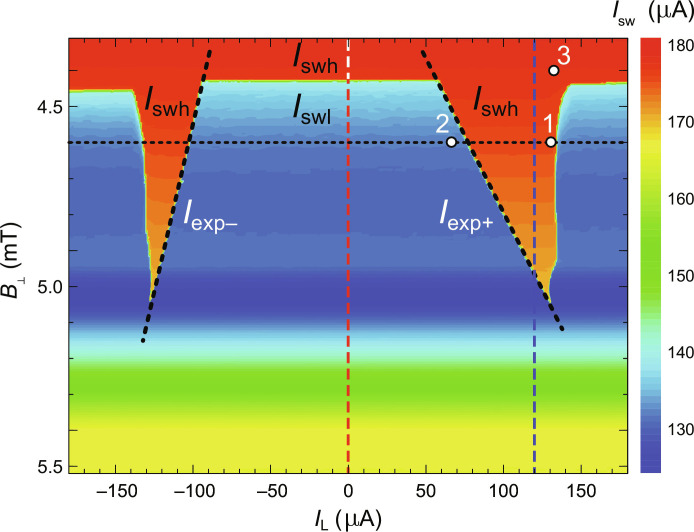
The as-received experimental vortex stability diagram *I*_sw_(*B*_⊥_, *I*_L_). Switching current dependence of the nanobridge on the applied perpendicular magnetic field *B*_⊥_ and the amplitude of the Lorentz pulse *I*_L_. It reveals range of magnetic fields in which vortex can be expelled with sufficiently high Lorentz pulse without switching the bridge. This range is marked by the existence of the two triangles in the diagram. The inner slopes of triangles (indicated with dashed lines) mark the minimum value of the Lorentz pulse necessary to expel the vortex *I*_exp_(*B*_⊥_). The outer edges correspond to the switching thresholds of the device, i.e., when *I*_L_ exceeds these thresholds, it necessarily switches the bridge and, thus, effectively works as the second reset pulse. The switching current (measured with the testing pulse) is low when it has to expel the vortex from the box and high if the box is in the Meissner state. The two cross sections of the diagram that are denoted with the vertical dashed lines are presented in Fig. 2B. The horizontal dashed line marks the cross section visible in Fig. 2C. The indicated points 1 to 3 refer to Fig. 4. The extended discussion of the vortex stability diagram is provided in fig. S7.

The *I*_sw_(*I*_L_) scan can be collected for various magnetic fields building the vortex stability diagram, i.e., the *I*_sw_(*B*_⊥_, *I*_L_) map (Fig. 3). The map shows two triangularly shaped regions of magnetic field and the Lorentz current pulse where expulsion of the vortex is possible without switching the junction.

### Dissipation due to a single vortex

Since the switching current of the bridge is uniquely related to the temperature of the box, both *I*_swh_ and *I*_swl_ can be converted into temperature with the aim of the *I*_sw_(*T*) calibration (see Materials and Methods and figs. S2 and S8). The resulting temperature difference Δ*T* = *T*(*I*_swl_) − *T*(*I*_swh_) = *T*_h_ − *T*_l_ corresponds to the instantaneous temperature increase due to the expulsion of the vortex out of the box. For the presented map, *T*_l_ = *T*_0_ = 400 mK and *T*_h_ ∼ 650 mK. Defining the volume of the SVB as Ω_B_ = *ta*^2^ (with *t* = 30 nm and *a* = 1 μm denoting the thickness of the superconducting box and the length of its side, respectively) and taking the dependence of the aluminum heat capacity *C*_p_(*T*) from literature (*41*) as a reasonable approximation, the measured Δ*T* yields

$$\Delta Q=\Omega_{B}\int_{0.4\;K}^{0.65\;K}‍C_{p}\left(T\right)\mathrm{dT}=4.3\cdot 10^{-19}\;J$$ (1)

as a calorimetric estimation of the released heat. This energy is equivalent to the absorption of a single photon of the visible light. In the studied range of parameters, we do not observe any substantial changes of this energy with magnetic field *B*_⊥_ or expulsion current *I*_exp_: The measured temperature rise after the vortex expulsion Δ*T* remains very similar, as it is evident from almost constant values of *I*_swh_ and *I*_swl_ visible as the red and blue regions in Fig. 3.

In the picture of the viscous flow of the vortex (*42*), the dissipation of energy in the superconducting box occurs as the vortex sweeps through a specific volume, causing the transformation of Cooper pairs into QPs (*14*, *43*, *44*). The volume within which the conversion occurs can be defined by the trajectory of the moving vortex, i.e., as $\Omega_{v}=\frac{1}{2}\mathrm{at}\xi$ , where ξ ≅ 150 nm is an estimate for the coherence length of the superconductor at 400 mK. Since the superconducting pairing only involves electrons at the surface of the Fermi sea, the number of QPs created in the process is *n*_qp_ = *g*(*E*_f_)Ω_v_Δ, where *g*(*E*_f_) is the density of states at the Fermi level and Δ = 1.76*k*_B_*T*_c_ = 200 μeV is the superconducting gap. The required excitation energy is roughly Δ*Q*_v_ = *n*_qp_Δ = 3.1 · 10^−19^ J and matches the magnitude of the dissipated heat Δ*Q* found in the experiment (see Materials and Methods for a complementary discussion).

### Thermal fingerprint of a single vortex

**Fig. 4.**
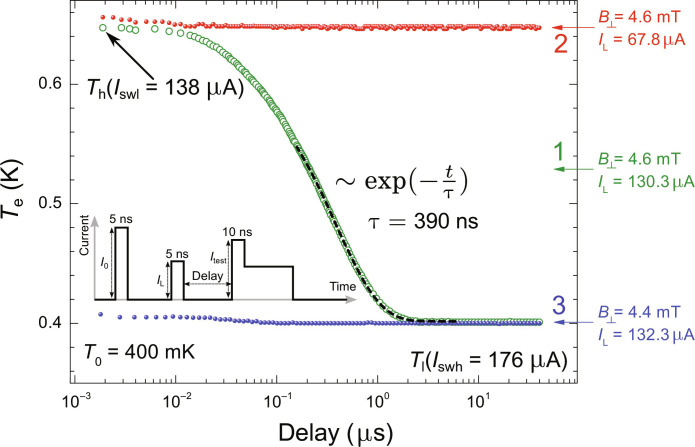
Experimental thermal dynamics of the SVB after expulsion of a single vortex. We apply the IL = 130.3 μA at *B*_⊥_ = 4.6 mT to get rid of the vortex and observe the following thermal transient of the box (curve 1). The other two curves are references revealing no electron temperature variation after the application of either a too low Lorentz pulse to expel the vortex (*I*_L_ = 67.8 μA, curve 2) or a too low magnetic field to trap the vortex after the application of the reset pulse (*B*_⊥_ = 4.4 mT, curve 3). In the case of the curve 2, the testing pulse itself expels the vortex. It results in the elevated value of the probed temperature, which is independent of the delay. The broken line, imposed on the curve 1, represents the exponential fit in the linear regime. The three points (*I*_L_, *B*_⊥_), corresponding to the three curves, are imposed on the vortex stability diagram in Fig. 3.

The excited QPs spread immediately in the SVB—the diffusion time across the box is of the order of 100 ps—and equilibrate with other electrons, which leads to the increase in the thermodynamic temperature of the box. Using the protocol of the nanosecond-resolving switching thermometry (*17*), we can measure the temporal relaxation profile of the box after expulsion of the vortex. It is accomplished by measuring the switching current of the bridge for various delays between the Lorentz and testing pulse (Fig. 4). The relaxation time in linear regime is 390 ns, in agreement with the thermal relaxation times for the aluminum nanowires studied by us in the earlier works, where either the switching of the nanobridge to the normal state (*17*) or the Joule heating of metallic island (*15*) was used to excite the QPs. The time is slightly smaller than expected from the electron-phonon relaxation channel alone due to the nonnegligible role of the QP diffusion along the leads and nonzero value of the magnetic field. The flat profile of the relaxation curve during first 10 ns, where relaxation has hardly started, allows us, however, to neglect the hot electron diffusion in the estimation of the dissipated energy ∆*Q*. Systematic studies of thermal relaxations triggered by expulsion of the vortex and measured for various *B*_⊥_ and *I*_L_ values are presented in figs. S3 to S5.

## DISCUSSION

Our experiment allows to trigger dissipation on demand in the self-limiting fundamental process: We have only one vortex that we can expel. One may envisage the demonstrated scheme as a convenient way to generate a limited and well-defined number of QPs in a superconducting structure, which enables the fully predictable and abrupt rise in the temperature of a superconductor without exceeding *T*_c_. This is in stark contrast to current-induced phase slips, often referred to as 1D vortices, which are indirectly observed in superconducting junctions and 1D nanowires near the critical current. Phase slips represent stochastic, and hence uncontrolled, dissipative events that eventually result in the transition of the samples to the normal state (*45*, *46*). It is expected that the dynamics of the expulsion process is of the order of a few tens of picoseconds (*14*)—the moving vortex provides a delta-like heating of the SVB. Thus, the expulsion itself is too fast to be observed experimentally, but it produces the measurable thermal trace due to finite lifetime of the excited QPs.

Similarly to the phase slip process, conveniently considered in the landscape of the tilted-washboard potential (*40*), the vortex expulsion may involve thermally stimulated jump over the Bean-Livingston energy barrier (Fig. 1). Both processes are stochastic in the narrow range of electric currents. The transition between the blue region (vortex not expelled with the Lorentz pulse) and the red region (vortex expelled with the Lorentz pulse) in the vortex stability diagram (Fig. 3), as seen across the *I*_exp_(*B*_⊥_) line, is very sharp. However, by using a finer resolution for the Lorentz pulse, we can accurately measure the current-dependent probability of the vortex escape (fig. S9). Alternatively to the thermal activation, the vortex expulsion may possibly proceed through macroscopic quantum tunneling, a phenomenon widely studied not only in superconducting junctions (*47*) and wires (*48*) but also in magnetic clusters (*49*).

Our work and that presented in (*13*) both show that a phase slip event or vortex crossing the nanowire is responsible for substantial local dissipation leading to massive generation of QPs. This is the effect that is a priori neglected in well-established orthodox models of superconducting transitions of 1D nanowires (*50*), in which numerous phase slips, either thermal (*51*, *52*) or quantum (*53*, *54*), are fluctuations of the order parameter responsible for a finite resistance below *T*_c_. Similarly, the local temperature increase due to dissipation is neglected in the Resistivity and Capacitively Shunted Junction (RCSJ) model (*40*), which, in many experimental works, is used to describe a finite voltage in a running state or in a phase diffusion regime of Josephson junctions. However, if a single initial phase slip produces large dissipation and increases the electron temperature, then it may lead to a stochastic or deterministic and more rapid phase-slip avalanche. It is because the phase slip rate for a given bias current is extremely sensitive to the value of the critical current, which, in turn, is sensitive to temperature (e.g., fig. S8). The overall dissipation then leads to the transition of the current-biased nanowire (*45*, *46*) or junction to the normal state, if the evacuation of heat to phonons or via QP diffusion cannot balance out the temperature rise due to the phase slippage or the vortex flow. On the other hand, when the heat sinking is efficient, like, presumably, it is in a case of a short nanobridge attached to wide pads or low–critical current tunnel junctions at not too low temperatures, the current-biased junction may be kept in a thermal steady state at an elevated temperature below *T*_c_. In general, our study calls for the confrontation of the existing escape theories with the thermal budget of widely tested junctions or nanowires, which is governed by the competition of phase slips producing excess QPs and heat sinking due to electron-phonon coupling and QP diffusion.

We demonstrate the operation of an SVB, in which vortex can be manipulated similarly to an electron in a Single Electron Box (*26*, *55*). Because of the engineered geometrical confinement, we are in position to single out just one vortex and treat it as a particle, which can be created (trapped) and annihilated (expelled) with pulses of electrical current. This feature combined with the fast time-resolving thermometry provides a comprehensive experimental insight into the physics of moving vortices. The expulsion of a single superconducting vortex with the current pulse from a mesoscopic sample produces dissipation at the level of 4 · 10^−19^ J. This is the energy necessary to turn all Cooper pairs into QPs on the path of the escaping vortex. The supreme level of control of the vortex state and small size makes the presented SVB an attractive device for memory and logical applications in the field of superconducting/vortex electronics. In this realization, three pulses in the presented experiment are responsible for initialization of the memory cell, write operation (executed with the Lorentz pulse), and readout (performed with the testing pulse). The SVB also exhibits the diode effect visible in the the vortex stability diagram. Our experimental platform is well suited for verifying the possibility of adiabatic manipulation of vortices, which is necessary to operate them as true quantum objects (*56*).

## MATERIALS AND METHODS

### Pulse protocol for probing and manipulating a single vortex and measuring electron temperature of the SVB

**Fig. 5.**
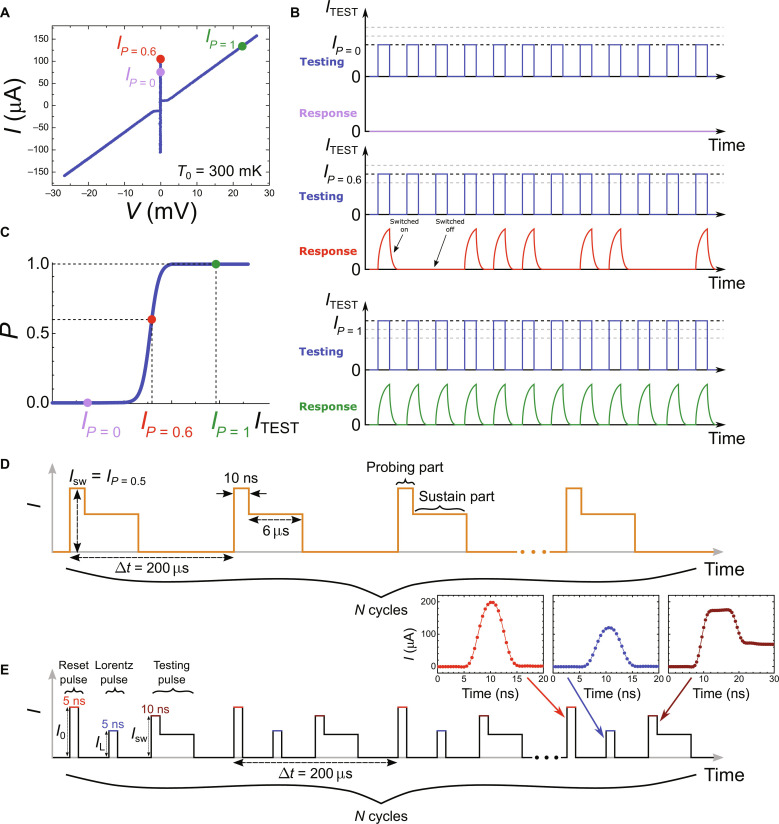
Measurement of the switching probability. (**A**) The current-voltage characteristics of a similar device to the one studied in this work. The supercurrent branch reveals the switching current of the nanobridge. (**B**) The fragments of trains of testing pulses, for three different amplitudes and their voltage response recorded on the device. The voltage drop appears when the nanobridge is switched to the normal state. (**C**) The dependence of nanobridge switching probability on the testing pulse amplitude, usually called the *S* curve. The three imposed points (*I*_TEST _for switching probability equal to 0, 0.6, and 1) correspond to the amplitudes of the pulses from (B). (**D**) The actual train of *N* testing pulses: Each pulse consists of a short probing part (testing the switching probability of the bridge), followed by a long sustain section (necessary to read out the switching events with low pass–filtered twisted pairs *f*_cut_ ≈ 80 kHz). (**E**) To manipulate the vortex, we add the two pulses to the each cycle of the standard probing protocol (D): reset pulse *I*_0_ to overheat the SVB above *T*_c_ and allow the field cooling of the SVB and Lorentz pulse *I*_L_ to exert force *F*_L_ on the vortex. The real shapes of the pulses, as visualized on the scope, are provided in the insets. The finite rising and trailing edges of the pulses are limited by the finite bandwidth of the arbitrary waveform generator (BW = 120 MHz). For on-chip imaging of the pulses, see appendix A of (*15*).

We perform our experiment in the dilution refrigerator. For aluminum bridges presented in this work, their switching current is very close to the critical current defining transition from the superconducting to the normal state. The method is based on testing of the bridge with train of *N* identical current pulses (*57*) to determine the switching probability *P*. Typically, *N* is equal 1000 or 10,000. If bridge switches *n* times, then the switching probability *P* = *n*/*N*. The testing pulses are repeated with period of Δ*t* = 200 μs guaranteeing a complete thermalization of the sample after each pulse. Consequently, to obtain a single value of probability, we need to record 200 ms to 2 s trace on the oscilloscope to count switching events. The measuring protocol is illustrated in Fig. 5. The switching current *I*_sw_ is defined as the one for which the switching probability of the bridge *P* is equal 0.5. It is found implicitly by adjusting the testing current amplitude with the bisection algorithm.

**Fig. 6.**
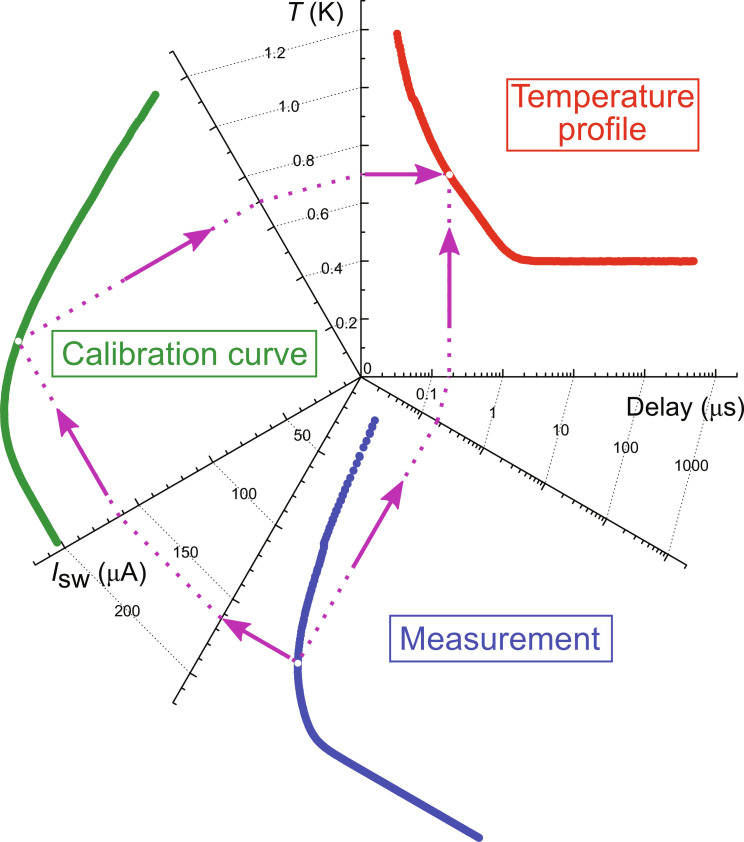
The conversion of the switching current to temperature. The transformation of the *I*_sw_(delay) into a temporal evolution of the temperature *T*(delay) with the application of the calibration curve *I*_sw_(*T*). An exemplary conversion for a single point is displayed with arrows. The *I*_sw_(*T*) curve bears information about values of the junction switching current at different electron temperatures. It is measured at fixed bath temperatures with electrons thermalized to the lattice.

The switching current of the bridge is sensitive to the local population of QPs, which, in turn, depends both on temperature and distribution of the Meissner screening currents. The first property makes the bridge a sensitive thermometer (*17*), and the second one allows for the detection of magnetic field and vortices, even if they are not expelled (*32*). The standard probing protocol is extended by the application of two additional pulses in each cycle, which precede the actual testing pulse (Figs. 1F and 5E). The first prepulse is so called reset pulse *I*_0_, for its amplitude is much higher (by ~30 to 50%) higher than the switching threshold of the bridge. Its role is to transit sample to the normal state and overheat the vortex box above *T*_c_ = 1.3 K. The subsequent cooling, taking place in the presence of applied magnetic field, allows to trap the vortex inside box, initializing the sample in a well-defined state. It takes around 20 ns for our structure to cool down back to *T*_c_ once the reset pulse is switched off (*17*). The reset pulse can, thus, be also thought of as a trapping pulse or initializing pulse. The second prepulse is intended to change the vortex state of the box without switching the bridge. It is called the Lorentz pulse *I*_L_, owing its name to the force it exerts on the vortex. By setting the time delay between the Lorentz and testing pulses, one can measure the temporal relaxation of the switching current of the bridge, which is a thermal consequence of the dissipative dynamics of a single vortex. For fast electron-electron interaction, the presence of excess QPs is interpreted as higher electron temperature. This allows us to convert the measured dependence of the switching current into the temporal profile of the electron temperature in the SVB (Fig. 6), which follows the expulsion of the vortex from the trap.

### Analysis of the dissipated energy

The dissipated heat comes from the Gibbs free energy difference between vortex and Meissner states Δ*G* = *G*_v_ − *G*_M_ and the work *W* done by the current source to expel the vortex. We can calculate the energy delivered from the current source during expulsion of the vortex with current *I*_exp_ and voltage *V* across the box by integrating instantaneous power over time window τ when vortex is being expelled

$$\Delta E_{s}\left(B_{⊥}\right)=\int_{0}^{\tau }‍VI_{\text{exp}}\;\mathrm{dt}$$ (2)

Using second Josephson relation, we get

$$\Delta E_{s}\left(B_{⊥}\right)=\int_{0}^{\tau }‍\frac{d\varphi }{\mathrm{dt}}\frac{\Phi_{0}}{2\pi }I_{\text{exp}}\;\mathrm{dt}=\frac{\Phi_{0}I_{\text{exp}}}{2\pi }\int_{0}^{\pi }‍d\varphi =\frac{\Phi_{0}I_{\text{exp}}}{2}$$ (3)

It is analogous formula to that for the energy dissipated in the phase slip event of the Josephson junction if to replace the critical current of the junction *I*_c_ with *I*_exp_ and notice that vortex leaving the box is equivalent to a half of phase slip, i.e., the phase across the box changes by value close to π. This energy ranges from *E*_s_ = 6 · 10^−20^ J to *E*_s_ = 1.2 · 10^−19^ J for *I*_exp_ = 60 μA to *I*_exp_ = 120 μA (cf. Fig. 3),which are the values 5 to 2.5 times smaller than the amount of the measured dissipated energy ∆*Q*. It suggests that in the observed process, the essential fraction of the dissipated energy comes from the Gibbs free energy difference between vortex and Meissner state of the box ∆*G* = *G*_v_ − *G*_M_, i.e., the vortex state corresponds to the local energy minimum but is not absolutely stable, as schematically presented in Fig. 1A for field *B*_⊥_ higher than *B*_0_ but smaller than *B_s_*. This interpretation would support claim (*30*, *31*, *58*) that in the cooled-down superconducting nanowire, magnetic field lines are first trapped when the condition for metastable equilibrium is met. Note that the initial (vortex) and final (Meissner) state of the box are obviously physically different. It is in contrast to the phase slip process in a junction or nanowire, in which the initial and final states are the same.

When increasing magnetic field, the state with vortex becomes energetically more favorable (the local energetic minimum becomes deeper), i.e., we get less heat from the free energy when transferring the SVB from the vortex to Meissner state (Δ*G* may turn negative at higher magnetic fields than these studied in the presented experiment). At the same time, we start to dissipate more energy from the current source. The energetic balance reads Δ*Q* = *W* + Δ*G*. In the studied range of parameters, we do not observe any substantial variation of Δ*Q* with *B*_⊥_ (see fig. S3). Moreover, once the vortex is expelled, we also see no difference in the amount of the dissipated energy Δ*Q* for the two polarities of the Lorentz pulse, although there is a noticeable difference in the expulsion threshold for them (see fig. S10).

## Supplementary Materials

### This PDF file includes:

Texts S1 and S2 Figs. S1 to S10 References
