# Supplementary material for: Quantum thermodynamics with a single superconducting vortex
Source: Sci Adv. 2024 Jul 31;10(31):eado4032. doi: 10.1126/sciadv.ado4032 (PMC11290525; doi:10.1126/sciadv.ado4032)
Supplement: Supplementary file 1 — Texts S1 and S2 Figs. S1 to S10 References [file sciadv.ado4032_sm.pdf]

Science Advances
AAAS

Supplementary Materials for

Quantum thermodynamics with a single superconducting vortex

Marek Foltyn *et al.*

Corresponding author: Maciej Zgirski, zgirski@ifpan.edu.pl

Sci. Adv. **10**, eado4032 (2024)
DOI: 10.1126/sciadv.ado4032

This PDF file includes:

Texts S1 and S2
Figs. S1 to S10
References

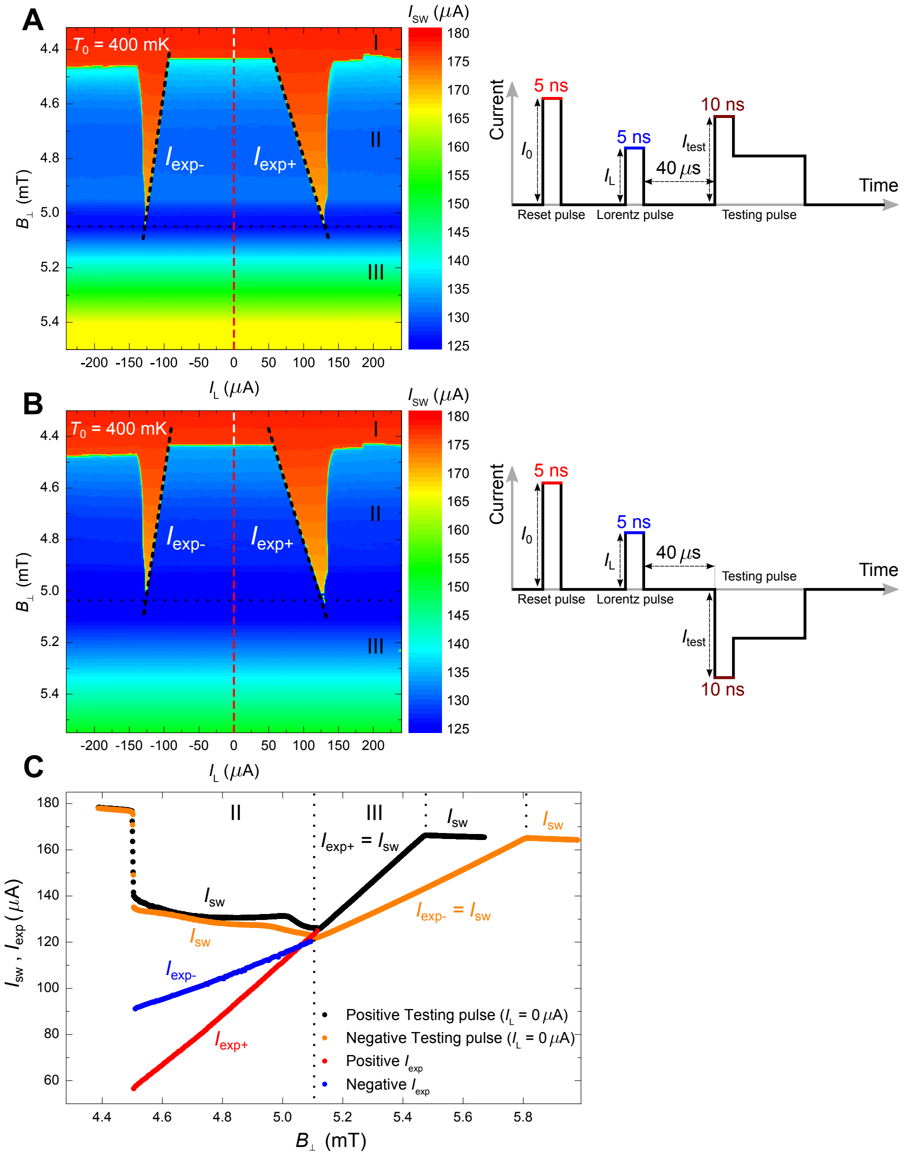

Figure S1: **Vortex diode effect.** (**A**) Vortex stability diagram collected for the positive testing pulses. (**B**) Vortex stability diagram collected for the negative testing pulses. The broken lines $I_L = 0$ and $I_L = I_{exp}$ indicate curves presented in panel C. (**C**) The switching current and the expulsion current dependencies on magnetic field. The two non-monotonous curves are the indicated cross-sections of the vortex diagrams at $I_L = 0$ collected for the positive and negative testing currents. The red and blue lines are the inner edges of the vortex stability diagrams which correspond to the onset of the vortex expulsion with the positive and negative Lorentz pulses I_L respectively (note that the positions of the inner edges does not depend on the polarity of the testing pulse, as expected). Importantly, the two curves for each polarity match together when crossing from zone II to III of the diagram creating

the two non-symmetric $I_{exp}(B_{\perp})$ almost linear dependencies for the two polarities. This evidences the diode effect. In the zone II it is the Lorentz pulse that expels the vortex (the switching current of the nanobridge is larger than the expulsion current, i.e. it is possible to expel the vortex without switching the bridge), in the zone III the testing pulse itself expels the vortex which leads to the switching of the junction (the switching current of the nanobridge after expelling the vortex is smaller than the expulsion current, i.e. expulsion of the vortex necessarily leads to the switching of the bridge).

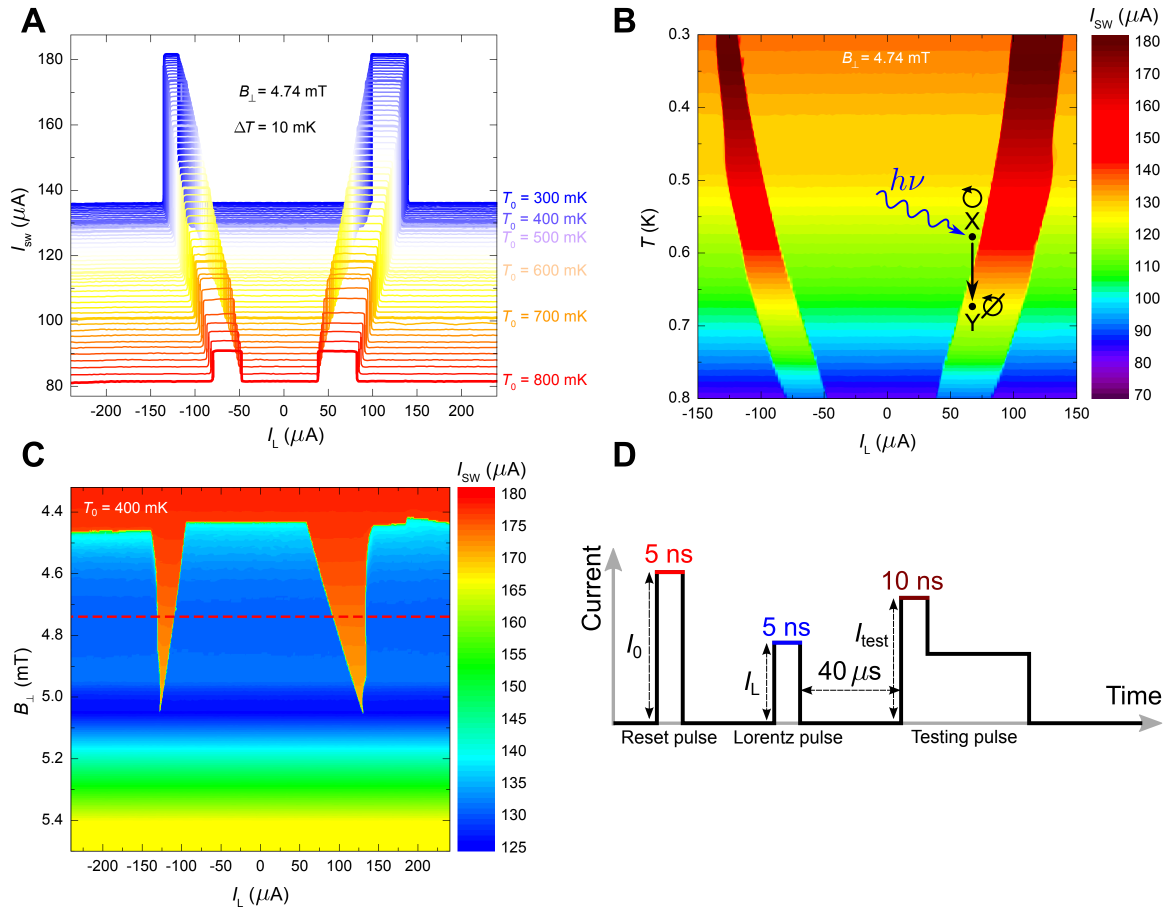

Figure S2: **Temperature dependence of the vortex expulsion current and the nanobridge switching current.** (**A**) Switching current of the nanobridge vs. the Lorentz pulse amplitude recorded for various temperatures at fixed magnetic fi eld. Inner edges of the high switching current plateaus mark the temperature dependent vortex expulsion current for the two polarities. Outer edges are the nanobridge switching thresholds. (**B**) The same data as in A presented as a contour plot $I_{sw}(T, I_L)$ illustrating the principle of the vortex amplifier for the enhanced photon detection. The vortex box biased with I_L just below the expulsion threshold, when heated by photon, will experience temperature rise (see the shift from point X to Y in panel B across the vortex expulsion edge). In point Y vortex is not stable any more. The dissipation assisting the expulsion provides much higher increase in temperature of the box than that initially caused by the photon absorption, and facilitates the detection process. (**C**) Vortex stability diagram from the main text recorded at 400 mK. The dashed line marks the one chosen $I_{sw}(I_L)$ dependence (for $B_\perp = 4.74$ mT) which can be also found in panels A and B

(for $T_0 = 400\,\mathrm{mK}$). (**D**) Testing protocol: for each Lorentz pulse amplitude I_L the presented sequence is repeated 1000 times to measure the switching probability of the nanobridge P. The amplitude of the testing pulse is adjusted with bisection algorithm to find the switching current defined as the one for which $P = 0.5$. For a given temperature (panels A and B) the low value of the switching current means the vortex expulsion on the rising slope of the testing pulse, and the high value of the switching current indicates the vortex expulsion during application of the Lorentz pulse.

Text S1 The Gibbs free energy of a single vortex

The total potential energy G of the vortex in the superconducting strip, shown in Fig. 1A, consists of four components (26,30):

1. The vortex core energy E_{core},

2. The energy of interaction of the vortex with its images:

$$E_{image} = -2\pi\rho \cdot ln \left[\frac{sin(\frac{\pi\xi}{w})}{sin(\frac{\pi y}{w})} \right] \tag{S1}$$

where ρ is the superconducting stiffness, ξ is the coherence length, y is the position across the strip and w is the strip width,

3. The interaction of the vortex with the magnetic field B:

$$E_{vB} = (2\pi)^2 \left(\frac{\rho B}{\Phi_0} \right) \left[\left(y - \frac{w}{2} \right)^2 - \left(\xi - \frac{w}{2} \right)^2 \right] \tag{S2}$$

4. The potential due to the Magnus force:

$$E_{Magnus} = \Phi_0 \left(\frac{J}{w} \right) \left(y - \frac{w}{2} \right) \tag{S3}$$

where J is the bias current.

Text S2 Essential length scales of a superconductor

A. Coherence length

The wavefunction changes at the scale of the coherence length, which in case of our SVB is estimated to be $\xi = 150\,\text{nm}$. To get this number we use the dirty limit formula for ξ, which is valid for $\xi_0 \gg l_{mean}$ (40):

$$\xi = 0.855\sqrt{\xi_0 l_{mean}} \tag{S4}$$

The elastic mean free path $l_{mean} \approx 20\,\text{nm}$ corresponds roughly to the grain size of the polycrystalline aluminum - see ref. 15 and the BCS value for clean aluminum is:

$$\xi_0 = \frac{\hbar v_F}{\pi \Delta_0} \cong 1.6\mu m \tag{S5}$$

where v_F is the Fermi velocity, Δ_0 is the superconducting gap at $T = 0\,\text{K}$. Using the definition of the diffusion constant in the form:

$$D = \frac{1}{3} v_F l_{mean} \tag{S6}$$

we can also cast the relation for the dirty limit coherence length in the form:

$$\xi = \sqrt{\frac{\hbar D}{\Delta_0}} \tag{S7}$$

This last form is for example used in ref. 59.

B. Penetration (screening) length

The magnetic field screening length (penetration depth) λ_L for a clean superconductor (i.e. bulk pure limit $\xi_0 \ll l_{mean}$) is given with the London relation:

$$\lambda_L^2(T) = \frac{m_e}{2\mu_0 n_s(T) e^2} \tag{S8}$$

where m_e is a single electron mass, e is an electron charge, n_s is the temperature dependent density of Cooper pairs, i.e. the number of paired electrons $n_{pe} = 2n_s$, μ_0 is the magnetic permeability of free space.

In the extreme dirty limit $\xi_0 \gg l_{mean}$ the effective screening length becomes (40):

$$\lambda_{eff}^2(T) = \lambda_L^2(T)\frac{\xi_0}{l_{mean}} \tag{S9}$$

Using the Drude formula for conductivity:

$$\sigma = \frac{ne^2 l_{mean}}{m_e v_F} \tag{S10}$$

with $n = 2n_s(0)$, we can also cast the relation for the dirty limit penetration depth in the form:

$$\lambda_{eff}^2(T=0) = \frac{\hbar}{\pi\mu_0\sigma\Delta_0} \tag{S11}$$

This last form is for example used in ref. 59.

The London penetration depth for aluminum at low temperature is $\lambda_L(0) \approx 15\,nm$. The dirty limit screening length is $\lambda_{eff}(0) = \lambda_L\sqrt{\frac{1600\,nm}{20\,nm}} = 135\,nm$.

C. Pearl length

The introduced dirty limit screening length λ_{eff} is further relaxed in thin films, i.e. in films with thickness $d \ll \lambda_{eff}$ (40,60). The proper length scale of the magnetic field penetration and screening currents becomes:

$$\Lambda = \frac{2\lambda_{eff}^2}{d} \tag{S12}$$

It is known in the literature as the Pearl length. It corresponds to the radius of the circular persistent current flowing around the vortex core. For the 30 nm thick aluminum film $\Lambda = 1.2\,\mu$m.

D. Ginzburg-Landau parameter κ

The value of the Ginzburg-Landau parameter $\kappa = \frac{\lambda(T)}{\xi(T)}$ defines the type of superconductivity we deal with. For $\kappa < \frac{1}{\sqrt{2}}$ it is type-I and existence of vortices is not possible. For $\kappa > \frac{1}{\sqrt{2}}$ vortices can enter the film and we have a type-II superconductor.

For SVB we define the effective $\kappa_{eff} = \frac{\Lambda}{\xi} = \frac{1200\,nm}{150\,nm} = 8 \gg \frac{1}{\sqrt{2}}$, which tells us that our structure is a type-II superconductor.

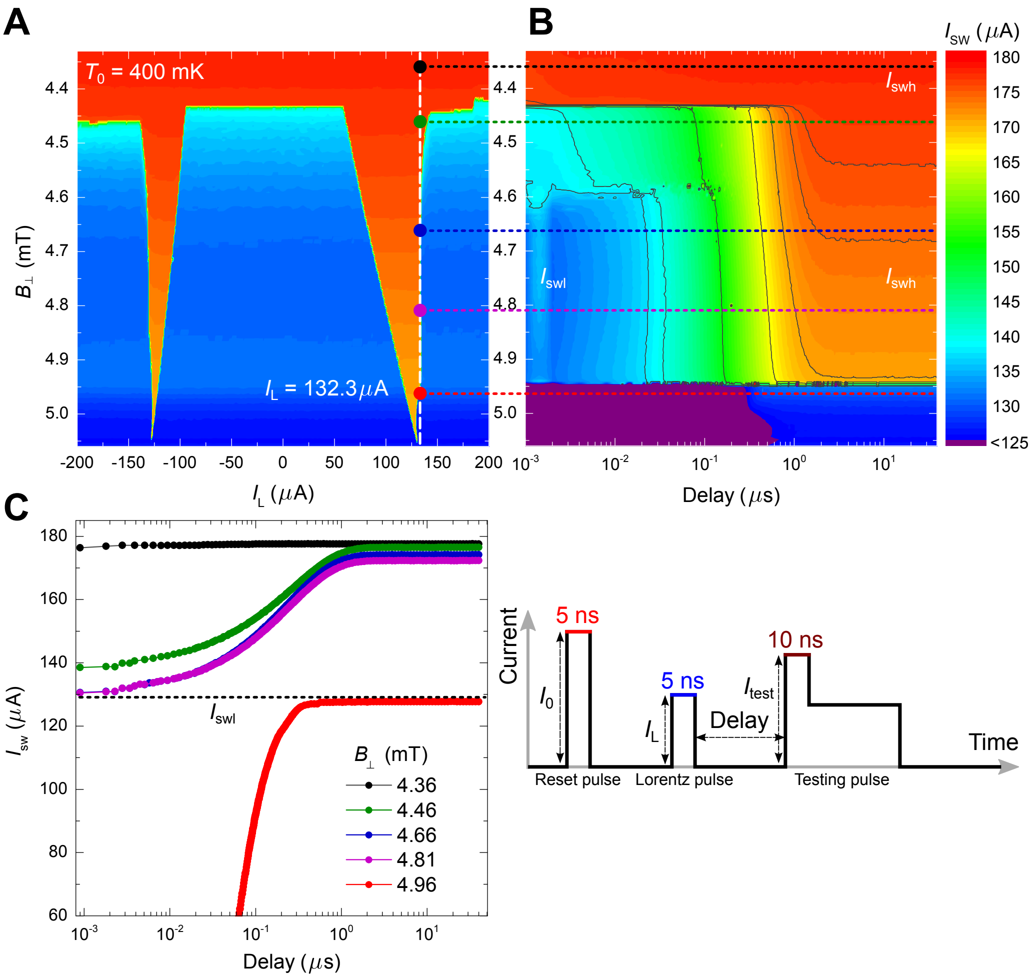

Figure S3: **Thermal relaxation after expulsion of the vortex – magnetic field study.** (**A**) Vortex stability diagram. Vertical dashed line shows the range of magnetic field for the relaxation experiment presented in panel B ($I_L = 132.3\,\mu$A). (**B**) Thermal relaxation map $I_{sw}(B, delay)$ collected for $I_L = 132.3\,\mu$A. Five horizontal dashed lines correspond to cross-sections of the map displayed in panel C. (**C**) Relaxation profiles for chosen values of magnetic field. We can distinguish 3 qualitatively different regions:

1. At low field (4.36 mT), vortex is absent in the structure and therefore the Lorentz pulse does not induce dissipation: we get the flat response.

2. Above the entry field, vortex can be expelled out of the structure without switching the junction. We observe the thermal relaxation of the switching current arising from the single vortex expulsion. The difference between the two branches of the relaxation curves ($B_\perp = 4.46$ mT and $B_\perp = 4.66$ mT)

may arise from not perfect fidelity in the initialization of the vortex state for smaller values of the magnetic field. As a result the relaxation curves recorded in this region ($B_{\perp} = 4.46\,\mathrm{mT}$) are the weighted averages of curves which would be measured with perfect fidelity and flat response (like that collected for $B_{\perp} = 4.36$ mT).

3. For too high magnetic field ($> 4.96\,\mathrm{mT}$), the Lorentz pulse not only expels the vortex but also necessarily leads to the switching of the junction. The structure is heated above T_c and once the Lorentz pulse is over, the box starts to cool-down trapping another vortex. When reading-out the bridge with testing pulse we have to expel the vortex, which leads to the additional dissipation: the thermal relaxation has an asymptote equal to the I_{swl} of the vortex stability diagram (see horizontal dashed line).

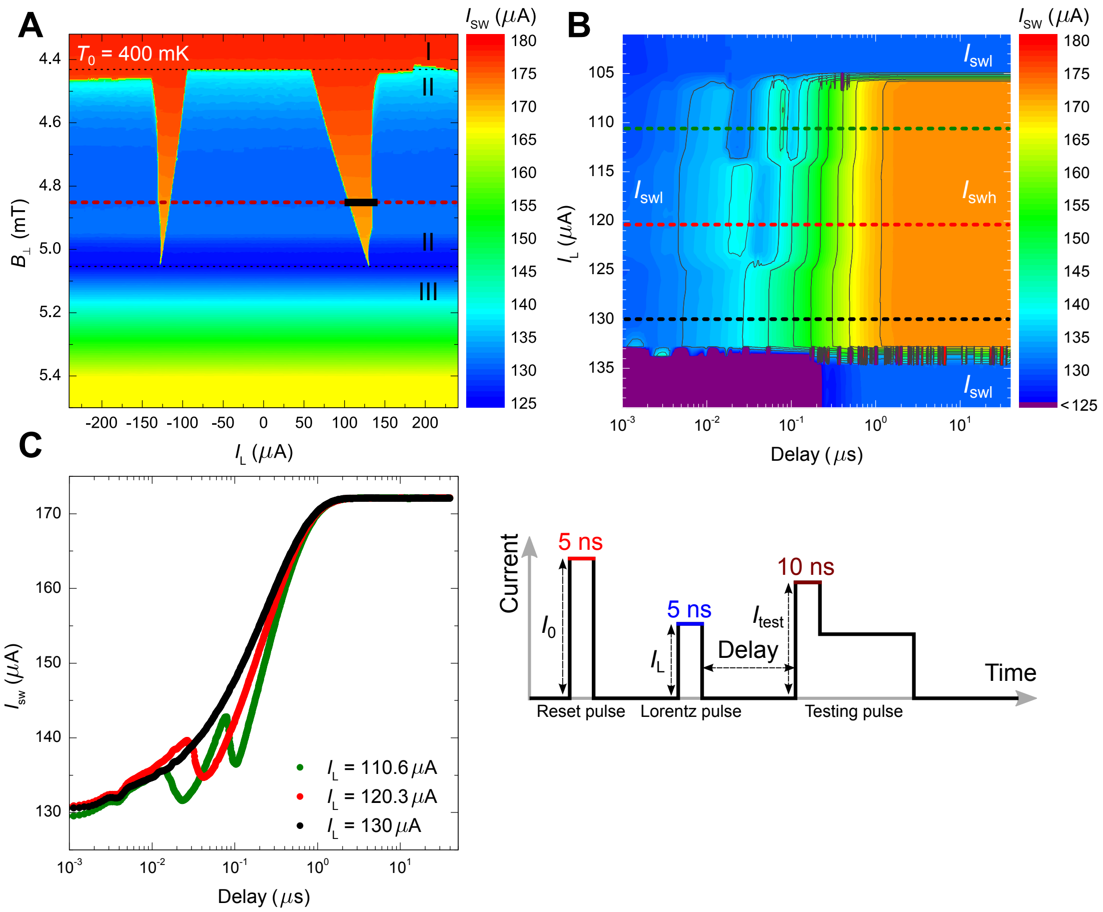

Figure S4: **Thermal relaxation after expulsion of the vortex – Lorentz pulse study (part I).** (**A**) Vortex stability diagram. Horizontal black thick line shows the range of the used Lorentz pulse amplitudes for the relaxation experiment presented in panel B ($B_\perp = 4.855$ mT). (**B**) Set of relaxation curves $I_{sw}(delay)$ collected for various I_L at fixed magnetic field $B_\perp = 4.855$ mT. We can distinguish three qualitatively different regions:

1. For $I_L < 105\,\mu$A we see no effect of the Lorentz pulse. It is too low to push out the vortex from the box. Testing pulse expels the vortex - it leads to heating, and then switches the junction. We observe low value of the switching current I_{swl}.

2. In the region $105\,\mu\text{A} < I_L < 133\,\mu$A vortex is expelled with the Lorentz pulse without switching the junction. We can measure the subsequent thermal relaxation with the testing pulse by varying its delay. The three horizontal dashed lines correspond to the relaxations displayed in panel C.

3. For $I_L > 133\,\mu$A the Lorentz pulse not only expels the vortex but also switches the junction and

overheats it above T_c. We can measure the subsequent thermal relaxation with the testing pulse as soon as the bridge enters back the superconducting state. Similarly to the red curve presented in Fig. S3C each time we test the bridge, we expel the vortex introducing another heat into the nanostructure. As a result the relaxation curve is shifted towards smaller values of the switching current. Its asymptote is equal to I_{swl}, indicating the switching process which immediately follows the expulsion of the vortex i.e. the vortex expulsion happens on the rising slope of the testing pulse.

(**C**) Relaxation profiles for the chosen values of the Lorentz pulse. All three curves show the same relaxation time. The profiles measured at the lower Lorentz pulse amplitudes show a non-monotonous behavior featuring one or two peaks. Only data measured for the Lorentz pulses whose amplitude is close to the switching threshold display a monotonous relaxation. We observe a sharp transition in the appearance of the relaxation curves as we increase the amplitude of the Lorentz pulse. Although not fully understood, we associate the non-monotonous relaxations with the dynamic trapping of quasiparticles in Andreev bound states of the nanobridge: when the nanostructure is cooled down some of superconducting channels in the bridge are blocked [such blocking effect is referred to as ”poisoning” in the literature (61,62)], which results in the suppression of the critical current of the nanobridge.

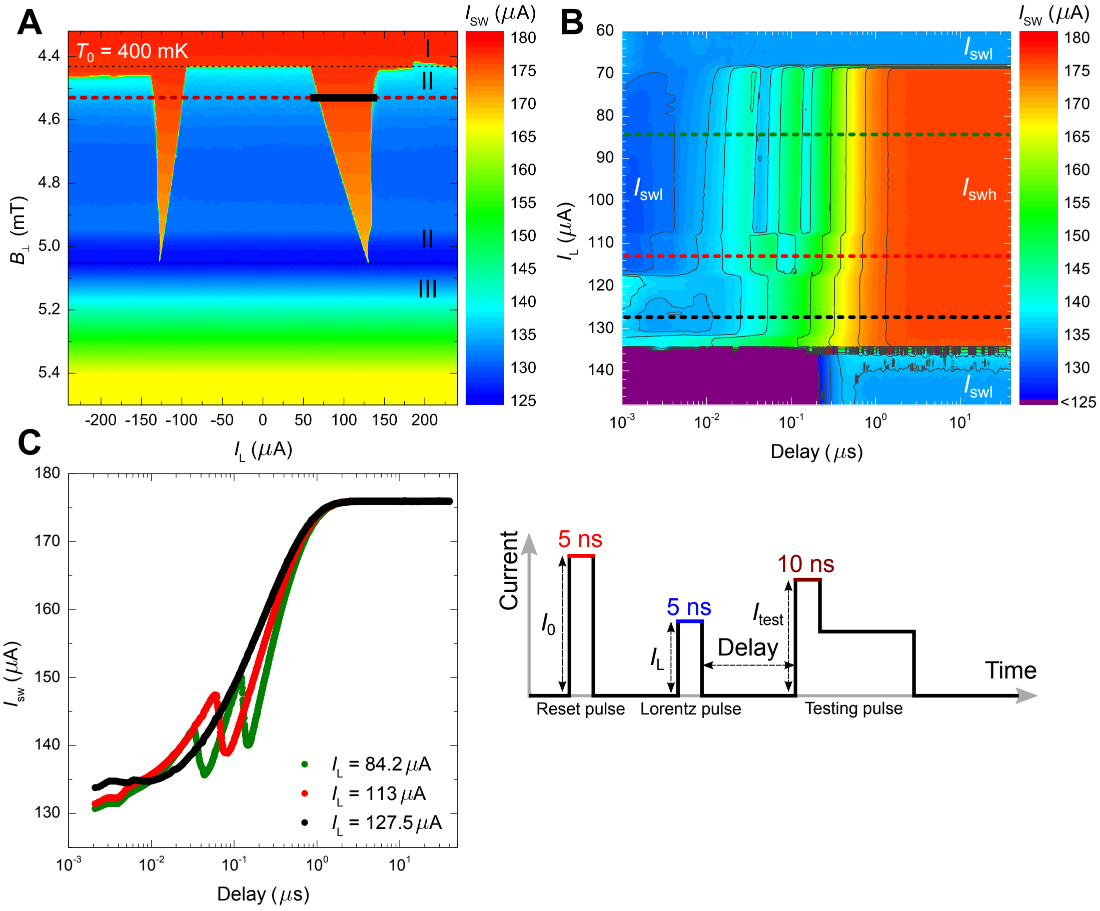

Figure S5: **Thermal relaxation after expulsion of the vortex – Lorentz pulse study (part II).** (**A**) Vortex stability diagram. Horizontal black thick line shows the range of the used Lorentz pulse amplitudes for the relaxation experiment presented in panel B ($B_\perp = 4.523\,\mathrm{mT}$). (**B**) Set of relaxation curves $I_{sw}(delay)$ collected for various I_L at fixed magnetic field $B_\perp = 4.523\,\mathrm{mT}$. We can distinguish three qualitatively different regions:

1. For $I_L < 67\,\mu\mathrm{A}$ we see no effect of the Lorentz pulse. It is too low to push out the vortex from the box. The testing pulse expels the vortex - it leads to the heating, and then switches the junction. We observe low value of the switching current I_{swl}.

2. In the region $67\,\mu\mathrm{A} < I_L < 133\,\mu\mathrm{A}$ vortex is expelled with the Lorentz pulse without switching the junction. We can measure the subsequent thermal relaxation with the testing pulse by varying its delay. The three horizontal dashed lines correspond to the relaxations displayed in panel C.

3. For $I_L > 133\,\mu\mathrm{A}$ the Lorentz pulse not only expels the vortex but also switches the junction.

We can measure the subsequent thermal relaxation from the normal state with the testing pulse, but each time we test the bridge we expel the vortex introducing another heat into the nanostructure. As a result the relaxation curves are shifted towards smaller values of the switching current. Their asymptote is equal to I_{swl}, indicating the switching process which immediately follows the expulsion of the vortex i.e. the vortex expulsion happens on the rising slope of the testing pulse.

(**C**) Relaxation profiles for the chosen values of the Lorentz pulse. All three curves show the same relaxation time. The profiles measured at the lower Lorentz pulse amplitudes show a non-monotonous behaviour featuring one or two peaks. Only data measured for the Lorentz pulses whose amplitude is close to the switching threshold display a monotonous relaxation. We observe a sharp transition in the appearance of the relaxation curves as we increase the amplitude of the Lorentz pulse. Although not fully understood, we associate the non-monotonous relaxations with the dynamic trapping of quasiparticles in Andreev bound states of the nanobridge: when the nanostructure is cooled-down some of the superconducting channels in the bridge are blocked [such blocking effect is referred to as ”poisoning” in the literature (61,62)], which results in the suppression of the critical current of the nanobridge.

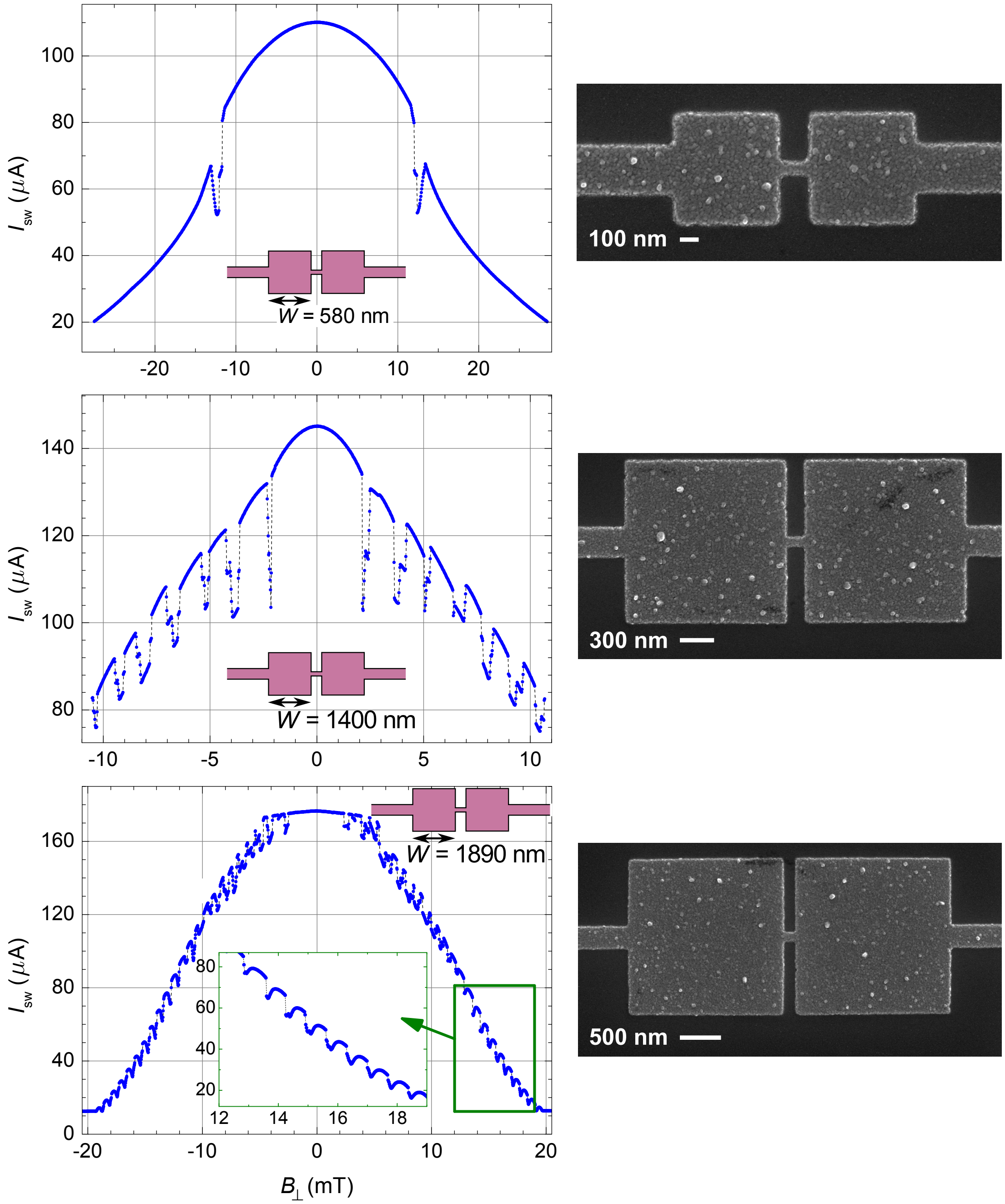

Figure S6: **Switching current of the nanobridge vs. perpendicular magnetic field $B_\perp$ for various vortex traps (T_0=400 mK).** Samples reveal drops in $I_{sw}(B_\perp)$ for field values where the entries of successive vortices are expected. The suppressed switching currents are measured right after expulsion of the vortex (or 2 vortices). The vortex is expelled on the rising slope of the testing pulse - it breaks Cooper pairs (rises temperature), which lowers the switching current of the nanobridge. The field for first vortex entry is well predicted with eq. (1) in Ref. 32. In between the dips vortices can

not be expelled with the current: the expulsion current scales with $B_\perp$ and becomes larger than the switching current. The majority of dips for sample $W = 1400\,\mathrm{nm}$ show two levels of the suppression: the deeper one corresponds to simultaneous expulsion of the two vortices, one from each trap; the shallower one is due to the expulsion of a single vortex from one trap when the vortices in the second trap become stable and can not be moved out by the current pulse. At higher magnetic fields the sample with $W = 1890\,\mathrm{nm}$ shows very regular I_{sw} oscillations (see inset). The geometry and morphology of the traps is provided in SEM images.

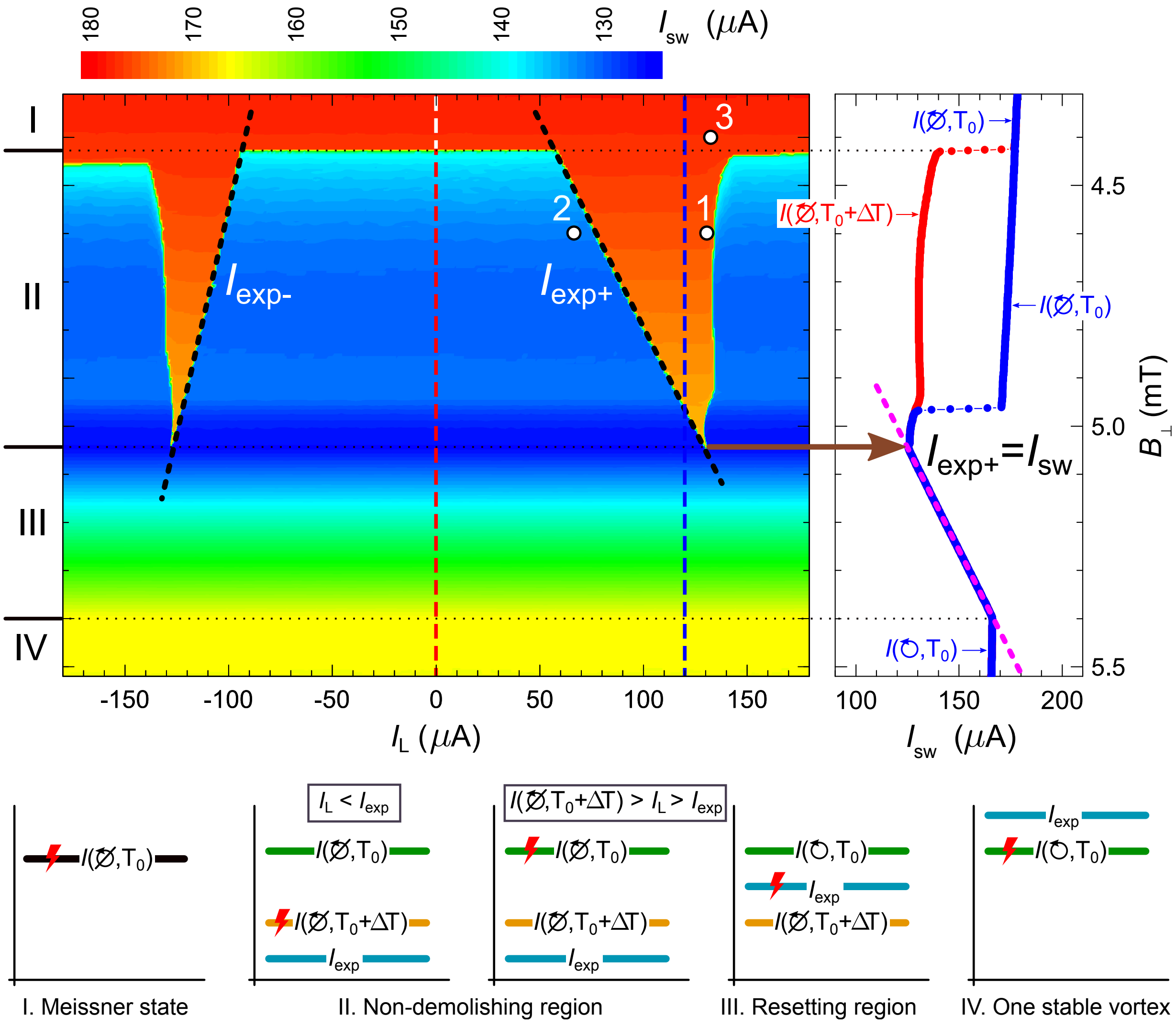

Figure S7: **The as-received experimental vortex stability diagram:** switching current dependence of the nanobridge on the applied magnetic field and the amplitude of the Lorentz pulse. It reveals four distinct regions in magnetic field: **I.** There is no vortex entry in the field-cooled sample after application of the reset pulse: the box remains in the Meissner state and the Lorentz pulse plays no role. **II.** Vortex is captured in the box just after the reset pulse (see Materials and Methods for details). It can be expelled with sufficiently high Lorentz pulse without switching the bridge. The zone of the Meissner state is thus extended into higher field values, what is observed as the two triangles in the diagram. The inner slopes of triangles (indicated with dashed lines) mark the minimum value of the Lorentz pulse necessary to expel the vortex $I_{exp}(B_\perp)$. The switching current (measured with the testing pulse) is low when it has to expel the vortex from the box, and high, if the box is in the

Meissner state - see also Fig. 2B. **III.** Vortex is captured in the box just after the reset pulse, as in region II. The required expulsion current I_{exp} is higher than the switching current after vortex has been expelled i.e. application of the Lorentz pulse capable of expelling the vortex necessarily leads to the switching of the bridge, providing the reset for the box. In this region the switching current is equal to the expulsion current - see the right panel. The line $I_{exp+}(B_\perp) = I_{sw}(B_\perp)$ is a continuation of $I_{exp+}(B_\perp)$ dependence from region II for larger magnetic fields (I_L and I_{sw} axes have the same unit revealing the same slope of the two pieces of $I_{exp+}(B_\perp)$ relation, see Fig. S1). **IV.** Vortex is captured in the box just after the reset pulse, as in region II and III. It is not possible to expel it because the required Lorentz pulse would need to be larger than the switching current of the bridge in the vortex state. The right figure contains two cross-sections of the main map which are denoted with the vertical dashed lines (the data are the same as those in Fig. 2B). The mutual relations between switching and expulsion currents in the four regions are illustrated schematically in the bottom panel (the loop and crossed loop respectively indicate the presence or absence of a vortex in the box at the very moment when the nanobridge switches). The switching levels are schematically indicated with the lightnings. The indicated points 1, 2, 3 refer to Fig. 4.

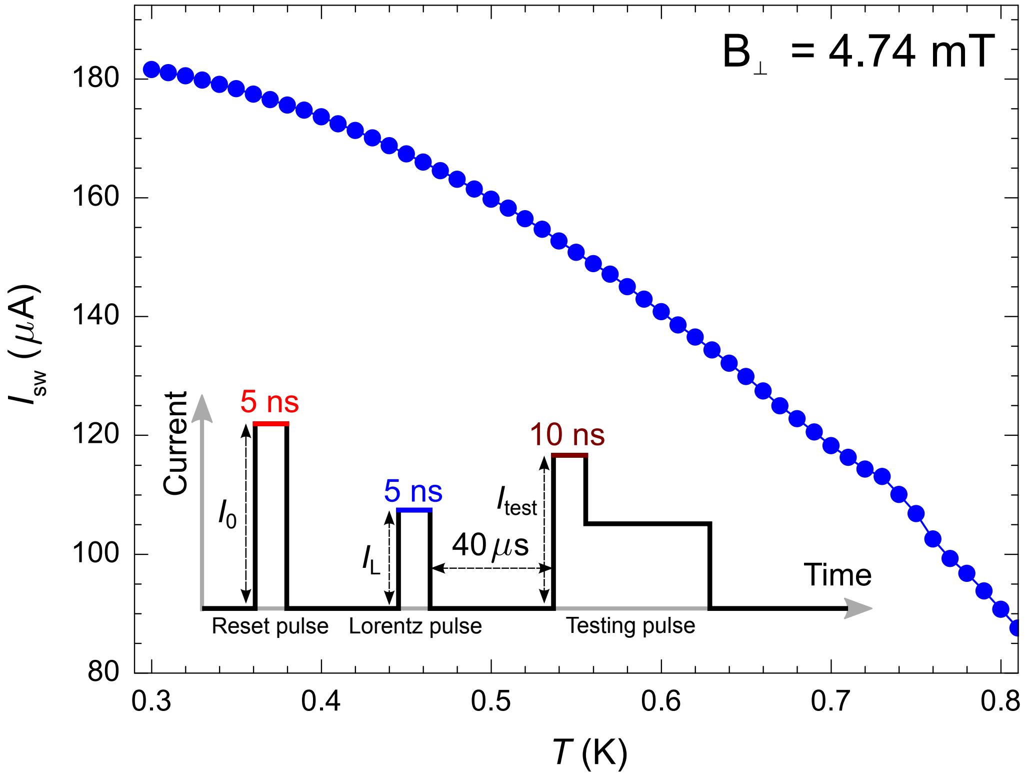

Figure S8: **Calibration curve $I_{sw}(T)$ in the state with no vortex.** Switching current of the nanobridge I_{sw} vs. the bath temperature T_0 measured after expulsion of the vortex in the thermally relaxed state for $B = 4.74$ mT (zone II in the vortex stability diagram - Fig. S7). The values are the plateaus visible in $I_{sw}(I_L)$ curves presented in panels A and B of the Fig. S2. The calibration curve is used to recalculate the measured $I_{sw}(delay)$ profiles into temporal dynamics of temperature $T(delay)$ after expulsion of the vortex.

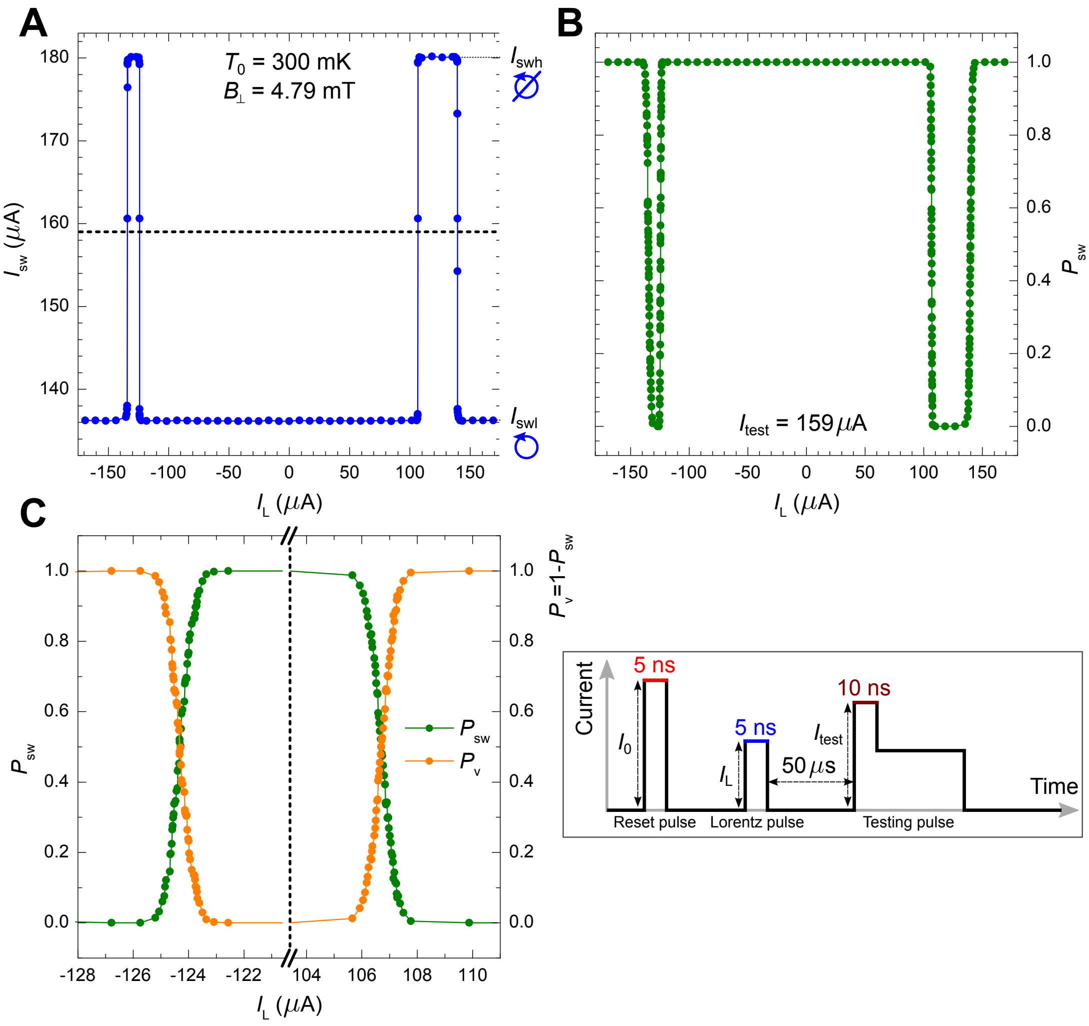

Figure S9: **Vortex expulsion probability.** (**A**) Switching current of the nanobridge measured with the testing pulse recorded as a function of the Lorentz pulse amplitude at constant magnetic field. I_{swl} and I_{swh} correspond to the presence and absence of the vortex in the box during testing, respectively. (**B**) Switching probability P_{sw} of the nanobridge measured as a function of the Lorentz pulse amplitude for the fixed testing current pulse $I_{test} = 159\ \mu$A at the constant magnetic field $B_\perp = 4.79$ mT. I_{test} is equal to $(I_{swl} + I_{swh})/2$ (see horizontal dashed line in panel A). The bridge necessarily switches during the testing pulse if the vortex is present in the box (i.e. it has not been expelled by the Lorentz pulse or has been trapped there upon cooling-down from the normal state after application of the too high Lorentz pulse), but it never switches if the vortex is absent (i.e. it has been expelled by the Lorentz pulse). (**C**) Magnified regions of the $P_{sw}(I_L)$ curve presented in panel B (left axis) and the resulting vortex expulsion probability $P_v = 1 - P_{sw}$(right axis).

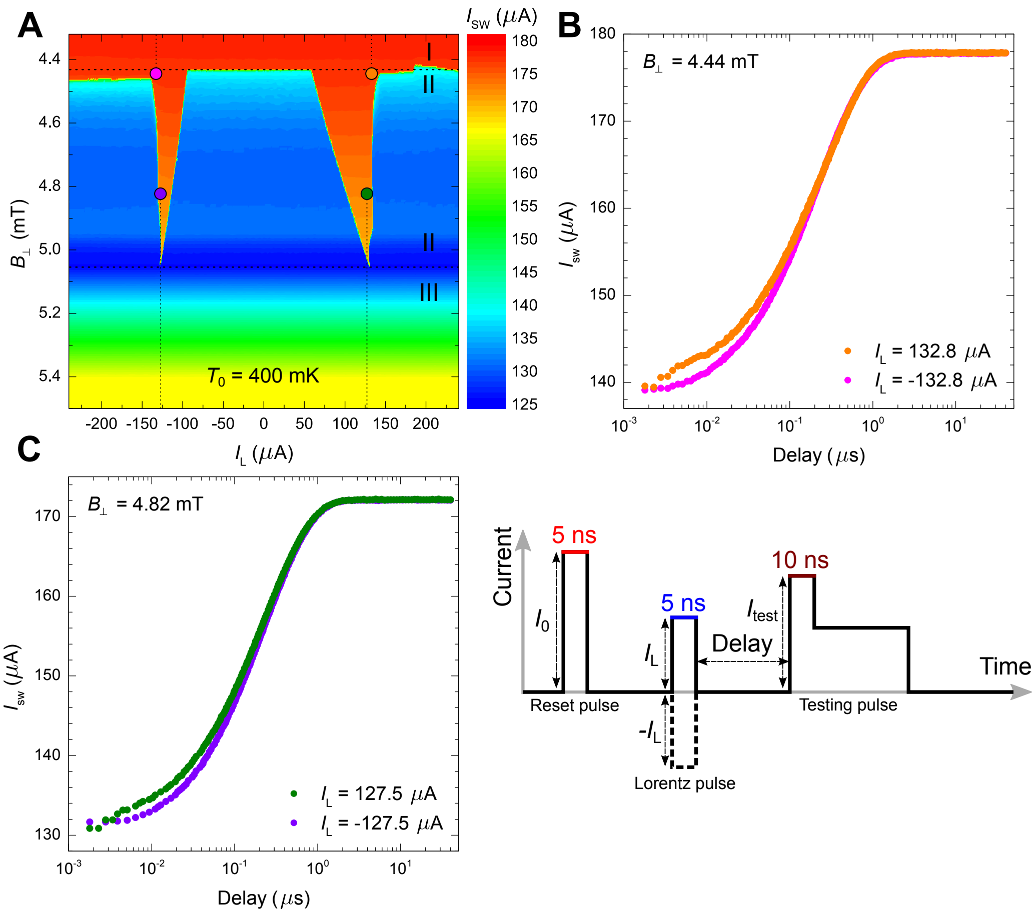

Figure S10: **Thermal dynamics of the SVB after vortex expulsion for the two polarities of the Lorentz pulse.** (**A**) Vortex stability diagram, measured at 400 mK. We indicate the two pairs of points, for which we record the thermal relaxations after expulsion of the vortex. (**B**) Relaxations of the switching current, measured at the two symmetric points of the vortex stability diagram ($I_L = 132.8\,\mu$A and $I_L = -132.8\,\mu$A at $B_\perp = 4.44\,$mT). Although the expulsion currents for the two polarities are significantly different (60 μA vs. 120 μA for positive vs. negative polarity respectively) the measured dissipation is the same, as indicated by the same suppression of the switching current. The vortex expulsion brings about $\Delta T \sim 250\,$mK temperature rise of the box in both cases. (**C**) Relaxations of the switching current measured at $I_L = 127.5\,\mu$A and $I_L = -127.5\,\mu$A at $B_\perp = 4.82\,$mT. Here, the expulsion currents for the two polarities are comparable, but significantly larger than for data presented in panel B. Nevertheless, the magnitude of dissipation revealed by all relaxation profiles remains very similar.

REFERENCES AND NOTES

1. J. P. Pekola, Towards quantum thermodynamics in electronic circuits. *Nat. Phys.* **11**, 118–123 (2015).

2. K. Schwab, E. A. Henriksen, J. M. Worlock, M. L. Roukes, Measurement of the quantum of thermal conductance. *Nature* **404**, 974–977 (2000).

3. M. Meschke, W. Guichard, J. P. Pekola, Single-mode heat conduction by photons. *Nature* **444**, 187–190 (2006).

4. M. Partanen, K. Y. Tan, J. Govenius, R. E. Lake, M. K. Mäkelä, T. Tanttu, M. Möttönen, Quantum-limited heat conduction over macroscopic distances. *Nat. Phys.* **12**, 460–464 (2016).

5. S. Jezouin, F. D. Parmentier, A. Anthore, U. Gennser, A. Cavanna, Y. Jin, F. Pierre, Quantum limit of heat flow across a single electronic channel. *Science* **342**, 601–604 (2013).

6. M. Banerjee, M. Heiblum, A. Rosenblatt, Y. Oreg, D. E. Feldman, A. Stern, V. Umansky, Observed quantization of anyonic heat flow. *Nature* **545**, 75–79 (2017).

7. M. Marin-Suarez, J. T. Peltonen, D. S. Golubev, J. P. Pekola, An electron turnstile for frequency-to-power conversion. *Nat. Nanotechnol.* **17**, 239–243 (2022).

8. Y. Utsumi, D. S. Golubev, M. Marthaler, K. Saito, T. Fujisawa, G. Schön, Bidirectional single-electron counting and the fluctuation theorem. *Phys. Rev. B* **81**, 125331 (2010).

9. J. V. Koski, A. Kutvonen, I. M. Khaymovich, T. Ala-Nissila, J. P. Pekola, On-chip Maxwell's demon as an information-powered refrigerator. *Phys. Rev. Lett.* **115**, 260602 (2015).

10. K. Maruyama, F. Nori, V. Vedral, Colloquium: The physics of Maxwell's demon and information. *Rev. Mod. Phys.* **81**, 1–23 (2009).

11. A. Bérut, A. Arakelyan, A. Petrosyan, S. Ciliberto, R. Dillenschneider, E. Lutz, Experimental verification of Landauer's principle linking information and thermodynamics. *Nature* **483**, 187–189 (2012).

12. D. Halbertal, J. Cuppens, M. B. Shalom, L. Embon, N. Shadmi, Y. Anahory, H. R. Naren, J. Sarkar, A. Uri, Y. Ronen, Y. Myasoedov, L. S. Levitov, E. Joselevich, A. K. Geim, E. Zeldov, Nanoscale thermal imaging of dissipation in quantum systems. *Nature* **539**, 407–410 (2016).

13. E. Gümüş, D. Majidi, D. Nikolić, P. Raif, B. Karimi, J. T. Peltonen, E. Scheer, J. P. Pekola, H. Courtois, W. Belzig, C. B. Winkelmann, Calorimetry of a phase slip in a Josephson junction. *Nat. Phys.* **19**, 196–200 (2023).

14. L. N. Bulaevskii, M. J. Graf, C. D. Batista, V. G. Kogan, Vortex-induced dissipation in narrow current-biased thin-film superconducting strips. *Phys. Rev. B* **83**, 144526 (2011).

15. M. Zgirski, M. Foltyn, A. Savin, A. Naumov, K. Norowski, Heat hunting in a freezer: Direct measurement of quasiparticle diffusion in superconducting nanowire. *Phys. Rev. Appl.* **14**, 044024 (2020).

16. A. V. Timofeev, C. P. García, N. B. Kopnin, A. M. Savin, M. Meschke, F. Giazotto, J. P. Pekola, Recombination-limited energy relaxation in a Bardeen-Cooper-Schrieffer superconductor. *Phys. Rev. Lett.* **102**, 017003 (2009).

17. M. Zgirski, M. Foltyn, A. Savin, K. Norowski, M. Meschke, J. Pekola, Nanosecond thermometry with Josephson junctions. *Phys. Rev. Appl.* **10**, 044068 (2018).

18. T. Golod, A. Iovan, V. M. Krasnov, Single Abrikosov vortices as quantized information bits. *Nat. Commun.* **6**, 8628 (2015).

19. N. Ligato, E. Strambini, F. Paolucci, F. Giazotto, Preliminary demonstration of a persistent Josephson phase-slip memory cell with topological protection. *Nat. Commun.* **12**, 5200 (2021).

20. I. Keren, A. Gutfreund, A. Noah, N. Fridman, A. Di Bernardo, H. Steinberg, Y. Anahory, Chip-integrated vortex manipulation. *Nano Lett.* **23**, 4669–4674 (2023).

21. D. Kalashnikov, V. Ruzhitskiy, A. Shishkin, I. Golovchanskiy, M. Kupriyanov, I. Soloviev, D. Roditchev, V. Stolyarov, Demonstration of a Josephson vortex-based memory cell with microwave energy-efficient readout. *Commun. Phys.* **7**, 88 (2024).

22. A. Engel, J. J. Renema, K. Il'in, A. Semenov, Detection mechanism of superconducting nanowire single-photon detectors. *Supercond. Sci. Technol.* **28**, 114003 (2015).

23. J. J. Renema, R. Gaudio, Q. Wang, Z. Zhou, A. Gaggero, F. Mattioli, R. Leoni, D. Sahin, M. J. A. de Dood, A. Fiore, M. P. van Exter, Experimental test of theories of the detection mechanism in a nanowire superconducting single photon detector. *Phys. Rev. Lett.* **112**, 117604 (2014).

24. L. Ceccarelli, D. Vasyukov, M. Wyss, G. Romagnoli, N. Rossi, L. Moser, M. Poggio, Imaging pinning and expulsion of individual superconducting vortices in amorphous MoSi thin films. *Phys. Rev. B* **100**, 104504 (2019).

25. L. Gottardi, S. Smith, "Transition-edge sensors for cryogenic x-ray imaging spectrometers" in *Handbook of X-ray and Gamma-ray Astrophysics* (Springer Nature Singapore, 2023), pp. 1–46.

26. D. Pekker, G. Refael, P. M. Goldbart, Weber blockade theory of magnetoresistance oscillations in superconducting strips. *Phys. Rev. Lett.* **107**, 017002 (2011).

27. T. Morgan-Wall, B. Leith, N. Hartman, A. Rahman, N. Marković, Measurement of critical currents of superconducting aluminum nanowires in external magnetic fields: Evidence for a Weber blockade. *Phys. Rev. Lett.* **114**, 077002 (2015).

28. C. P. Bean, J. D. Livingston, Surface barrier in type-II superconductors. *Phys. Rev. Lett.* **12**, 14–16 (1964).

29. K. K. Likharev, The formation of a mixed state in planar semiconductor films. *Radiophys. Quantum Electron.* **14**, 722–727 (1971).

30. G. M. Maksimova, Mixed state and critical current in narrow semiconducting films. *Phys. Solid State* **40**, 1607–1610 (1998).

31. G. Stan, S. B. Field, J. M. Martinis, Critical field for complete vortex expulsion from narrow superconducting strips. *Phys. Rev. Lett.* **92**, 097003 (2004).

32. M. Foltyn, K. Norowski, M. Wyszynński, A. de Arruda, M. Milosševicć, M. Zgirski, Probing confined vortices with a superconducting nanobridge. *Phys. Rev. Appl.* **19**, 044073 (2023).

33. M. Pannetier-Lecoeur, C. Fermon, Determination of the energy dissipation of a vortex in a superconductor using a giant magnetoresistive sensor. *Phys. Rev. B* **72**, 180501 (2005).

34. L. Embon, Y. Anahory, Ž. Jelić, E. O. Lachman, Y. Myasoedov, M. E. Huber, G. P. Mikitik, A. V. Silhanek, M. V. Milosševicć, A. Gurevich, E. Zeldov, Imaging of super-fast dynamics and flow instabilities of superconducting vortices. *Nat. Commun.* **8**, 85 (2017).

35. O. V. Dobrovolskiy, D. Y. Vodolazov, F. Porrati, R. Sachser, V. M. Bevz, M. Y. Mikhailov, A. V. Chumak, M. Huth, Ultra-fast vortex motion in a direct-write Nb-C superconductor. *Nat. Commun.* **11**, 3291 (2020).

36. Y. B. Kim, C. F. Hempstead, A. R. Strnad, Flux-flow resistance in type-II superconductors. *Phys. Rev.* **139**, A1163–A1172 (1965).

37. S. Field, J. Witt, F. Nori, X. Ling, Superconducting vortex avalanches. *Phys. Rev. Lett.* **74**, 1206–1209 (1995).

38. E. Altshuler, T. H. Johansen, Colloquium: Experiments in vortex avalanches. *Rev. Mod. Phys.* **76**, 471–487 (2004).

39. A. Lara, F. G. Aliev, V. V. Moshchalkov, Y. M. Galperin, Thermally driven inhibition of superconducting vortex avalanches. *Phys. Rev. Appl.* **8**, 034027 (2017).

40. M. Tinkham, *Introduction to Superconductivity* (Dover Publications, 2004).

41. N. E. Phillips, Heat capacity of aluminum between 0.1°K and 4.0°K. *Phys. Rev.* **114**, 676–685 (1959).

42. J. Bardeen, M. J. Stephen, Theory of the motion of vortices in superconductors. *Phys. Rev.* **140**, A1197–A1207 (1965).

43. A. I. Larkin, Y. N. Ovchinnikov, Nonlinear conductivity of superconductors in the mixed state. *Zh. Eksp. Teor. Fiz.* **68**, 1915–1927 (1975).

44. M. Golosovsky, M. Tsindlekht, D. Davidov, High-frequency vortex dynamics in $YBa_2Cu_3O_7$. *Supercond. Sci. Technol.* **9**, 10.1088/0953-2048/9/1/001 (1996).

45. M. Sahu, M.-H. Bae, A. Rogachev, D. Pekker, T.-C. Wei, N. Shah, P. M. Goldbart, A. Bezryadin, Individual topological tunnelling events of a quantum field probed through their macroscopic consequences. *Nat. Phys.* **5**, 503–508 (2009).

46. M. Zgirski, M. Foltyn, A. Savin, K. Norowski, Stochastic thermal feedback in switching measurements of a superconducting nanobridge caused by overheated electrons and phonons. *Phys. Rev. B* **104**, 014506 (2021).

47. J. Clarke, A. N. Cleland, M. H. Devoret, D. Esteve, J. M. Martinis, Quantum mechanics of a macroscopic variable: The phase difference of a Josephson junction. *Science* **239**, 992–997 (1988).

48. M. Zgirski, K.-P. Riikonen, V. Touboltsev, K. Arutyunov, Size dependent breakdown of superconductivity in ultranarrow nanowires. *Nano Lett.* **5**, 1029–1033 (2005).

49. W. Wernsdorfer, E. Bonet Orozco, K. Hasselbach, A. Benoit, D. Mailly, O. Kubo, H. Nakano, B. Barbara, Macroscopic quantum tunneling of magnetization of single ferrimagnetic nanoparticles of barium ferrite. *Phys. Rev. Lett.* **79**, 4014–4017 (1997).

50. M. Zgirski, "Experimental study of fluctuations in ultra-narrow superconducting nanowires," thesis, Department of Physics, University of Jyväskylä (2008).

51. J. E. Lukens, R. J. Warburton, W. W. Webb, Onset of quantized thermal fluctuations in "one-dimensional" superconductors. *Phys. Rev. Lett.* **25**, 1180–1184 (1970).

52. R. S. Newbower, M. R. Beasley, M. Tinkham, Fluctuation effects on the superconducting transition of tin whisker crystals. *Phys. Rev. B* **5**, 864–868 (1972).

53. C. N. Lau, N. Markovic, M. Bockrath, A. Bezryadin, M. Tinkham, Quantum phase slips in superconducting nanowires. *Phys. Rev. Lett.* **87**, 217003 (2001).

54. M. Zgirski, K.-P. Riikonen, V. Touboltsev, K. Y. Arutyunov, Quantum fluctuations in ultranarrow superconducting aluminum nanowires. *Phys. Rev. B* **77**, 054508 (2008).

55. H. Grabert, M. H. Devoret, *Single Charge Tunneling: Coulomb Blockade Phenomena in Nanostructures* (Nato Science Series, 1992).

56. A. Wallraff, A. Lukashenko, J. Lisenfeld, A. Kemp, M. V. Fistul, Y. Koval, A. V. Ustinov, Quantum dynamics of a single vortex. *Nature* **425**, 155–158 (2003).

57. M. Foltyn, M. Zgirski, Gambling with superconducting fluctuations. *Phys. Rev. Appl.* **4**, 024002 (2015).

58. J. R. Clem, Vortex exclusion from superconducting strips and SQUIDs in weak perpendicular ambient magnetic fields. *Bull. Am. Phys. Soc.* **43**, 411 (1998).

59. A. Anthore, H. Pothier, D. Esteve, Density of states in a superconductor carrying a supercurrent. *Phys. Rev. Lett.* **90**, 127001 (2003).

60. J. Pearl, Current distribution in superconducting films carrying quantized fluxoids. *Appl. Phys. Lett.* **5**, 65–66 (1964).

61. M. Zgirski, L. Bretheau, Q. Le Masne, H. Pothier, D. Esteve, C. Urbina, Evidence for long-lived quasiparticles trapped in superconducting point contacts. *Phys. Rev. Lett.* **106**, 257003 (2011).

62. E. M. Levenson-Falk, F. Kos, R. Vijay, L. Glazman, I. Siddiqi, Single-quasiparticle trapping in aluminum nanobridge Josephson junctions. *Phys. Rev. Lett.* **112**, 047002 (2014).
